# Subunit-Dependent Surface Mobility and Localization of NMDA Receptors in Hippocampal Neurons Measured Using Nanobody Probes

**DOI:** 10.1523/JNEUROSCI.2014-22.2023

**Published:** 2023-06-28

**Authors:** Stepan Kortus, Kristyna Rehakova, Martin Klima, Marharyta Kolcheva, Marek Ladislav, Emily Langore, Petra Barackova, Jakub Netolicky, Anna Misiachna, Katarina Hemelikova, Jana Humpolickova, Dominika Chalupska, Jan Silhan, Martina Kaniakova, Barbora Hrcka Krausova, Evzen Boura, Martin Zapotocky, Martin Horak

**Affiliations:** ^1^Institute of Experimental Medicine of the Czech Academy of Sciences, 142 20 Prague 4, Czech Republic; ^2^Institute of Organic Chemistry and Biochemistry of the Czech Academy of Sciences, 166 10 Prague 6, Czech Republic; ^3^Department of Physiology, Faculty of Science, Charles University in Prague, 128 43 Prague 2, Czech Republic; ^4^Institute of Physiology of the Czech Academy of Sciences, 142 20 Prague 4, Czech Republic

**Keywords:** excitatory synapse, GluN subunit, glutamate receptor, lateral diffusion, live microscopy, mammalian neuron

## Abstract

NMDA receptors (NMDARs) are ionotropic glutamate receptors that play a key role in excitatory neurotransmission. The number and subtype of surface NMDARs are regulated at several levels, including their externalization, internalization, and lateral diffusion between the synaptic and extrasynaptic regions. Here, we used novel anti-GFP (green fluorescent protein) nanobodies conjugated to either the smallest commercially available quantum dot 525 (QD525) or the several nanometer larger (and thus brighter) QD605 (referred to as nanoGFP-QD525 and nanoGFP-QD605, respectively). Targeting the yellow fluorescent protein-tagged GluN1 subunit in rat hippocampal neurons, we compared these two probes to a previously established larger probe, a rabbit anti-GFP IgG together with a secondary IgG conjugated to QD605 (referred to as antiGFP-QD605). The nanoGFP-based probes allowed faster lateral diffusion of the NMDARs, with several-fold increased median values of the diffusion coefficient (*D*). Using thresholded tdTomato-Homer1c signals to mark synaptic regions, we found that the nanoprobe-based *D* values sharply increased at distances over 100 nm from the synaptic edge, while *D* values for antiGFP-QD605 probe remained unchanged up to a 400 nm distance. Using the nanoGFP-QD605 probe in hippocampal neurons expressing the GFP-GluN2A, GFP-GluN2B, or GFP-GluN3A subunits, we detected subunit-dependent differences in the synaptic localization of NMDARs, *D* value, synaptic residence time, and synaptic–extrasynaptic exchange rate. Finally, we confirmed the applicability of the nanoGFP-QD605 probe to study differences in the distribution of synaptic NMDARs by comparing to data obtained with nanoGFPs conjugated to organic fluorophores, using universal point accumulation imaging in nanoscale topography and direct stochastic optical reconstruction microscopy.

**SIGNIFICANCE STATEMENT** Our study systematically compared the localization and mobility of surface NMDARs containing GFP-GluN2A, GFP-GluN2B, or GFP-GluN3A subunits expressed in rodent hippocampal neurons, using anti-green fluorescent protein (GFP) nanobodies conjugated to the quantum dot 605 (nanoGFP-QD605), as well as nanoGFP probes conjugated with small organic fluorophores. Our comprehensive analysis showed that the method used to delineate the synaptic region plays an important role in the study of synaptic and extrasynaptic pools of NMDARs. In addition, we showed that the nanoGFP-QD605 probe has optimal parameters for studying the mobility of NMDARs because of its high localization accuracy comparable to direct stochastic optical reconstruction microscopy and longer scan time compared with universal point accumulation imaging in nanoscale topography. The developed approaches are readily applicable to the study of any GFP-labeled membrane receptors expressed in mammalian neurons.

## Introduction

NMDA receptors (NMDARs) are ionotropic glutamate receptors that play an essential role in excitatory neurotransmission. Functional NMDARs are heterotetramers composed of two GluN1 subunits combined with two GluN2 (GluN2A through GluN2D) and/or GluN3 (GluN3A and GluN3B) subunits (Sanz-Clemente et al., 2013; Paoletti et al., 2013; Hansen et al., 2021). The major GluN2 subunits expressed in the postnatal forebrain change during development; specifically, the GluN2B subunit is abundant at birth but its expression then decreases, whereas GluN2A subunit expression increases for several days after birth (Monyer et al., 1994; Sheng et al., 1994; Wenzel et al., 1997). Interestingly, the GluN3A subunit is strongly expressed during the narrow window correlated with intense synaptogenesis (Wong et al., 2002; Pérez-Otaño et al., 2006; Kehoe et al., 2014).

The number and subtypes of NMDARs at the cell surface are tightly regulated at several levels (Horak et al., 2014), including their surface mobility and lateral diffusion between synaptic and extrasynaptic regions (Dupuis and Groc, 2020). Various single molecule-tracking techniques have been developed to measure the surface mobility of membrane receptors (Bouzigues et al., 2007; Giannone et al., 2010), including the use of either fluorescent organic dyes (e.g., ATTO 647N) or fluorescent semiconductor nanocrystals called quantum dots (QDs), both of which are conjugated to a protein such as an antibody that recognizes a specific surface receptor (Bouzigues et al., 2007; Giannone et al., 2010; Lee et al., 2017). Considering the extremely narrow (∼20 nm) synaptic cleft, the obvious advantage of fluorescent organic dyes is their relatively small diameter (∼1 nm; Dikić et al., 2012); however, their use is limited by their short time frame (on the order of seconds) because of rapid photobleaching and by the low number of photons emitted by a single fluorophore (Giannone et al., 2010). On the other hand, QDs have bright, stable fluorescence and are resistant to photobleaching, allowing for longer tracking times (on the order of minutes; Bouzigues et al., 2007). The emission wavelength of a given QD depends on the size of its core, but the actual size of a QD is larger than fluorescent organic dyes as they must be coated to be soluble in aqueous media, making the diameter >10 nm (Sheung et al., 2018). Similarly, the protein part of the probe affects the overall size of the probe. For example, a conventional IgG antibody molecule is considerably larger in size (∼14.5 × 8.5 × 4 nm, with a molecular weight of ∼150 kDa) than the smallest currently known antigen-binding proteins, V_H_H nanobodies (length, ∼4 nm; diameter, ∼2.5 nm; molecular weight range, ∼12–15 kDa; Kubala et al., 2010; Asaadi et al., 2021).

To date, only a limited number of studies examined the difference in surface mobility between NMDAR subtypes in neurons; moreover, these studies addressed only the differences between GluN2A-containing and GluN2B-containing NMDARs and predominantly used primary and secondary IgG antibodies conjugated to QD655 (e.g., anti-GluN2A, anti-GluN2B, and anti-FLAG antibodies; Groc et al., 2006; Papouin et al., 2012; Dupuis et al., 2014; Ferreira et al., 2017). These studies reported the following: (1) GluN2A-containing NMDARs have a lower diffusion coefficient (*D*) compared with GluN2B-containing NMDARs in both synaptic and extrasynaptic regions (Groc et al., 2006); (2) GluN2A-containing NMDARs are more stable than GluN2B-containing NMDARs at synapses (Groc et al., 2006); and (3) the mobility of synaptic GluN2B-containing NMDARs is reduced in the presence of d-serine because of reduced interaction with the postsynaptic scaffold protein PSD-95 (Ferreira et al., 2017). We previously studied the effect of *N*-glycosylation on the surface mobility of GluN3A-containing NMDARs using a primary anti-green fluorescent protein (GFP) IgG antibody combined with a QD605-labeled secondary IgG antibody, but we were unable to distinguish between synaptic and extrasynaptic QD trajectories (Skrenkova et al., 2018). Here, we used a comprehensive approach to investigate the surface mobility of NMDARs in synaptic and extrasynaptic regions of hippocampal neurons using several nanobody probes.

## Materials and Methods

### Lentiviruses

We used a previously described lentivirus expressing a yellow fluorescent protein (YFP)-tagged rat GluN1-1a subunit (NCBI reference sequence: NM_017010.2; Lichnerova et al., 2015). We also prepared FHUGW lentiviral vectors expressing GFP-tagged rat GluN2A (NCBI reference sequence: NM_012573.4), GluN2B (NCBI reference sequence: NM_012574.1), and GluN3A subunit (NCBI reference sequence: NM_001198583.2; in all constructs, we introduced the A206K point mutation in GFP to reduce GFP dimerization; Zacharias et al., 2002). To label excitatory synapses, we used a lentivirus expressing tdTomato-Homer1c (BL-1034) or custom-made Cre-P2A-tdTomato-Homer1c (Cre-tdTomato-Homer1c). All lentiviruses used in this project were generated at the Viral Core Facility at Charité–Universitätsmedizin Berlin (Charité–Berlin University of Medicine).

### Preparation of transfected human embryonic kidney 293 cells

Human embryonic kidney 293 (HEK293) cells were cultured in Opti-MEM I medium containing 5% (v/v) fetal bovine serum (Thermo Fisher Scientific) and transfected using PolyMag reagent (OZ Biosciences) using a total of 0.9 μg of plasmids expressing GFP (to identify successfully transfected cells), the rat version of the GluN1-4a subunit (NCBI reference sequence: NM_001270610.1); together with the GFP-GluN2A or GluN2A (NCBI reference sequence: NM_012573.4); GFP-GluN2B or GluN2B (NCBI reference sequence: NM_012574.1); and/or GFP-GluN3A or GluN3A (NCBI reference sequence: NM_001198583.2) subunits. For recording, the transfected HEK293 cells were trypsinized and cultured at lower density on poly-l-lysine-coated glass coverslips.

### Preparation of cultured hippocampal neurons

All animal experiments were performed in accordance with the guidelines established by the European Union Directive 2010/63/EU, the European Council Directive dated November 24, 1986 (86/609/EEC), and animal care guidelines approved by the Animal Care Committee of the Institute of Experimental Medicine of the Czech Academy of Sciences. Primary cultures of hippocampal neurons were prepared from embryonic day 18 Wistar rat embryos or C57BL/6N-GluN2A^flox/flox^/GluN2B^flox/flox^ double conditional knock-out (cKO) mice (hereafter referred to as cKO-GluN2A/GluN2B mice; crossbred in our internal animal facility from the GluN2A^flox/flox^ and GluN2B^flox/flox^ cKO mice; Gray et al., 2011) as described previously (Kolcheva et al., 2021). In brief, the hippocampi were removed, submerged in papain (Worthington Biochemical), diluted in Earle's balanced salt solution containing 10 mm HEPES, and incubated for 15 min in a water bath at 37°C. The cells were then dissociated and plated in plating medium consisting of minimum essential medium supplemented with 10% (v/v) heat-inactivated horse serum, N2 supplement (1×), 1 mm sodium pyruvate, 20 mm d-glucose, 25 mm HEPES, and 1% penicillin-streptomycin. After 2 h, the plating medium was replaced with growth medium composed of Neurobasal medium with 2% B-27 supplement, 0.5 mm GlutaMAX, and 1% penicillin-streptomycin. The cells were grown at a density of approximately 2 × 10^4^ cells/cm^2^ in a 35 mm Petri dish with a 20 mm microwell (catalog #D35-20–1.5H, Cellvis) coated with poly-l-lysine (Merck). Half of the growth medium volume was replaced every 3–4 d with fresh medium. Lentiviral infection was performed after 7 d *in vitro* 7 (DIV7), and infected neurons were used for experiments at DIV14 to DIV16. Unless otherwise stated, all chemicals were purchased from Thermo Fisher Scientific.

### Preparation of GFP nanobody QD525 and GFP nanobody QD605 probes

The bacterial expression vector encoding GFP nanobody (nanoGFP; catalog #49172, Addgene) was a gift from Brett Collins (The Institute for Molecular Bioscience, Queensland). NanoGFP including the C-terminal hexahistidine purification tag was expressed in *Escherichia coli* strain BL21 DE3 NiCo (New England Biolabs) in ZY autoinduction medium. Bacterial cells were harvested by centrifugation and lysed in lysis buffer consisting of 50 mm Tris-HCl, pH 8; 300 mm NaCl; 30 mm imidazole; and 10% glycerol. The lysate was then incubated with HisPur Ni-NTA Superflow Agarose (Thermo Fisher Scientific) for 30 min, and the agarose beads were washed thoroughly with washing buffer consisting of 50 mm Tris-HCl, pH 8; 300 mm NaCl; and 20 mm imidazole. The nanoGFP protein was eluted using elution buffer consisting of 50 mm Tris-HCl, pH 8; 300 mm NaCl; and 300 mm imidazole, and then purified further using size exclusion chromatography on a HiLoad 16/60 Superdex 75 Prep Grade Column (Cytiva) in PBS, pH 7.4. The molecular weight and protein purity of nanoGFP were verified by performing SDS-PAGE and matrix-assisted laser desorption/ionization analysis. The purified protein was concentrated to 5 mg/ml, aliquoted, flash frozen in liquid nitrogen, and stored at −80°C. The purified nanoGFP was conjugated to commercially available QD525 and QD605 (Q21541MP and Q21501MP, respectively; Thermo Fisher Scientific) in accordance with the manufacturer protocol. In brief, QD525 and QD605 were transferred to PBS using 100 kDa ultrafiltration units, transferred to glass vials, conjugated with the amino-amine crosslinker bis(sulfosuccinimidyl)suberate (BS3; Thermo Fisher Scientific), and purified by removing excess crosslinker using size-exclusion chromatography on a Superdex 75 10/300 GL Column (Cytiva) in PBS. Both QDs were then incubated with a 40-fold molar excess of nanoGFP for 2 h, quenched with 50 mm glycine, pH 6.5, for 15 min, and again purified to remove excess nanoGFP using size-exclusion chromatography on a Superdex 75 10/300 GL Column (Cytiva) in PBS. Finally, the purified nanoGFP-QD525 and nanoGFP-QD605 conjugates were concentrated to 1 μm and stored at 4°C.

### Preparation of nanoGFP-ATTO647N and nanoGFP-AF647 probes

The purified nanoGFP was conjugated to commercially available Alexa Fluor 647 NHS ester (catalog #AF647, Thermo Fisher Scientific) and ATTO 647N NHS Ester (catalog #ATTO647N, ATTO-TEC) in accordance with the protocols of the manufacturers. In brief, the fluorescent dyes were dissolved in dimethylsulfoxide at 10 mg/ml, added to nanoGFP in PBS at a molar ratio of 1:3 (nanobody/dye), and incubated for 1 h. The reactions were quenched with 100 mm hydroxylamine, pH 8.5. The conjugates were then separated from excess dyes by size-exclusion chromatography using a Superdex 75 10/300 GL Column (Cytiva) in PBS. Finally, the purified conjugates were concentrated to 1 mg/ml, aliquoted, and stored at 4°C. The degrees of labeling (i.e., the average number of fluorescent dye molecules per protein molecule; nanoGFP:ATTO647N, 0.42; nanoGFP:AF647, 1.53) were determined by spectrophotometry using the known spectral characteristics of the respective dyes.

### Measurement of the QD hydrodynamic radius using fluorescence correlation spectroscopy

Fluorescence correlation spectroscopy (FCS) was performed using an confocal microscope (model SP8, Leica) equipped with a high numerical aperture (NA) 1.2, 60× water-immersion objective. The QD525 and QD605 probes were excited with 488 nm and 561 nm light, respectively, with the laser power at the back aperture of the objective set at <2 µW. The fluorescent light passed through the pinhole and was focused on an HyD detector. Because the concentration of the QDs was 10 nm and the light was collected from a diffraction-limited volume of ∼1 fl, the fluorescence fluctuations provide information regarding the diffusion rate (*D*) and therefore the hydrodynamic radius of the QDs.

The collected signal was temporally autocorrelated using custom-written scripts in MATLAB and fitted using the following mathematical model, which assumes a single population of diffusing particles:

(1) G(t)=1+1N(1+tτD)−1(1+S−2tτD)−12,

where *N* stands for the number of diffusing particles in the focal volume, τ*_D_* is the mean transition time, and *S* is the parameter that structurally characterizes the focal volume. τ*_D_* is related to the diffusion coefficient as follows: $\omega^{2}=4D\tau_{D}$, where ω is the lateral radius of the confocal volume. ω and *S* were determined by independent calibration using a fluorescent dye with a known *D* value. We used ATTO488-carboxylic acid (4.0 ± 0.1 × 10^−6^ cm^2^/s) and Rhodamine B (4.27 ± 0.04 × 10^−6^ cm^2^/s) for the 488 and 561 nm laser lines, respectively (Dertinger et al., 2011). The obtained *D* value was subsequently transformed to hydrodynamic radius (*R*_H_) using the Stokes–Einstein equation, as follows:

$$R_{\text{H}}=\frac{k_{\text{B}}T}{6\pi \eta D},$$

where *k*_B_ is the Boltzmann constant, *T* is absolute temperature, and η is viscosity.

### Transmission electron microscopy

The 400 mesh carbon-coated Cu grids were glow discharged for 30 s at 0.3 bar and 30 mA on GloQube (Quorum Technologies). To perform transmission electron microscopy (TEM) with negatively stained QD probes, 3 µl of a 100 nm solution with QD probes were applied onto the grids and incubated for 1 min at room temperature. The grid was then immersed in 15 µl of 2% uranyl acetate and, after 30 s, was blotted with filter paper; this step was repeated twice. Finally, the grids were dried in air for 10 min (Douglas et al., 2018). The samples were loaded into a transmission electron microscope (model JEM-2100Plus, JEOL) and imaged at a magnification of 60,000× and a pixel (px) size of 1.939 Å/px. The particles corresponding to the QDs were measured using the ImageJ program (Schneider et al., 2012).

### Microscopy with QD probes

Before measuring the surface mobility of QD-labeled YFP-GluN/GFP-GluN subunits, the neurons were washed with Neurobasal medium (Thermo Fisher Scientific) containing 1.25% (w/v) casein (Vector Laboratories) or 1% (w/v) bovine serum albumin (BSA; Merck). The neurons were then incubated for 7 min with nanoGFP-QD525 or nanoGFP-QD605 probes diluted to a final concentration of ∼1 nm in Neurobasal medium containing casein or BSA; alternatively, some neurons were incubated for 7 min with rabbit anti-GFP antibody (1:2000; Merck), followed by incubation for 7 min with anti-rabbit IgG antibody conjugated to QD605 (hereafter called antiGFP-QD605; 1:10,000; Thermo Fisher Scientific). The neurons were then washed with Neurobasal medium and placed in imaging solution (IS) containing the following (in mm): 160 NaCl, 2.5 KCl, 2 CaCl_2_, 1 MgCl_2_, 10 HEPES, and 10 glucose, pH 7.3 at 37°C. The surface mobility of QD-labeled YFP-GluN/GFP-GluN subunits was measured in live hippocampal neurons using a modified version of our recently published strategy (Skrenkova et al., 2018); specifically, using a DeltaVision OMX imaging platform equipped with a pco.edge 5.5 sCMOS camera, the QDs were excited at 395/29 nm using a DAPI excitation filter, and emission was detected using FITC 528/48 (for QD525) or Alexa Fluor 609/37 (for QD605) emission filters. Typically, a total of 1201 consecutive frames were recorded at a frame rate of 50 ms and an exposure time of 35 ms. The media and IS were preheated to 37°C before use, and all microscopy images were obtained within 30 min after labeling with the respective QD probes. The imaging dish was placed in a chamber that was preheated to 37°C. The temperature in the field of view was controlled for each experiment using a temperature sensor in the solution, and imaging started after the temperature in the dish had stabilized at 37°C (usually within 5 min after exchanging the imaged dish). An Olympus 60× oil-immersion lens (NA 1.42) heated to 37°C was used for imaging. As a default, the OMX microscope was calibrated for GFP at 23°C using immersion oil with a refraction index of 1.512. For surface mobility, immersion oil with a refraction index of 1.1518 was chosen after measuring the point spread function (PSF) at 37°C, emission at 609 nm, and ∼1 µm depth to minimize aberrations caused by the combination of the oil lens and the water-based solution. When indicated, the QD-labeled neurons were fixed in 4% paraformaldehyde (PFA; Merck) in PBS for 20 min and then washed with PBS. The built-in OMX software was used to register the multichannel images (i.e., the GFP, QD525 or QD605, and tdTomato images) in accordance with the manufacturer instructions. Calibration registration efficiency and the PSF were verified using prepared calibration slides containing fluorescent TetraSpeck Microspheres emitting at all wavelengths used in these experiments (Thermo Fisher Scientific).

### Image analysis

The QDs were tracked and assembled into trajectories as positions over time using custom-written MATLAB script with the freeware tracking engine TrackMate (Tinevez et al., 2017). The TrackMate parameters were chosen empirically to achieve the best tracking performance for a given probe as follows: QD605: subpixel localization = true; radius = 0.25 µm, threshold = 2.5, median filter = true, spot quality threshold = 10; QD525: subpixel localization = true, radius = 0.15 µm, threshold = 10, median filter = true, spot quality threshold = 12; track assembly parameters for QD525 and QD605: linkage maximum distance = 0.25 µm; gap closure maximum distance = 0.5 µm; maximum interframe gap = 10 frames. The QD trajectories generated using TrackMate and corrected for drift were combined with the tdTomato-Homer1c and YFP-GluN/GFP-GluN signals, packaged, and saved as a single structured MATLAB (.mat) file containing all necessary information for further processing.

### Drift correction

**Figure 1.**
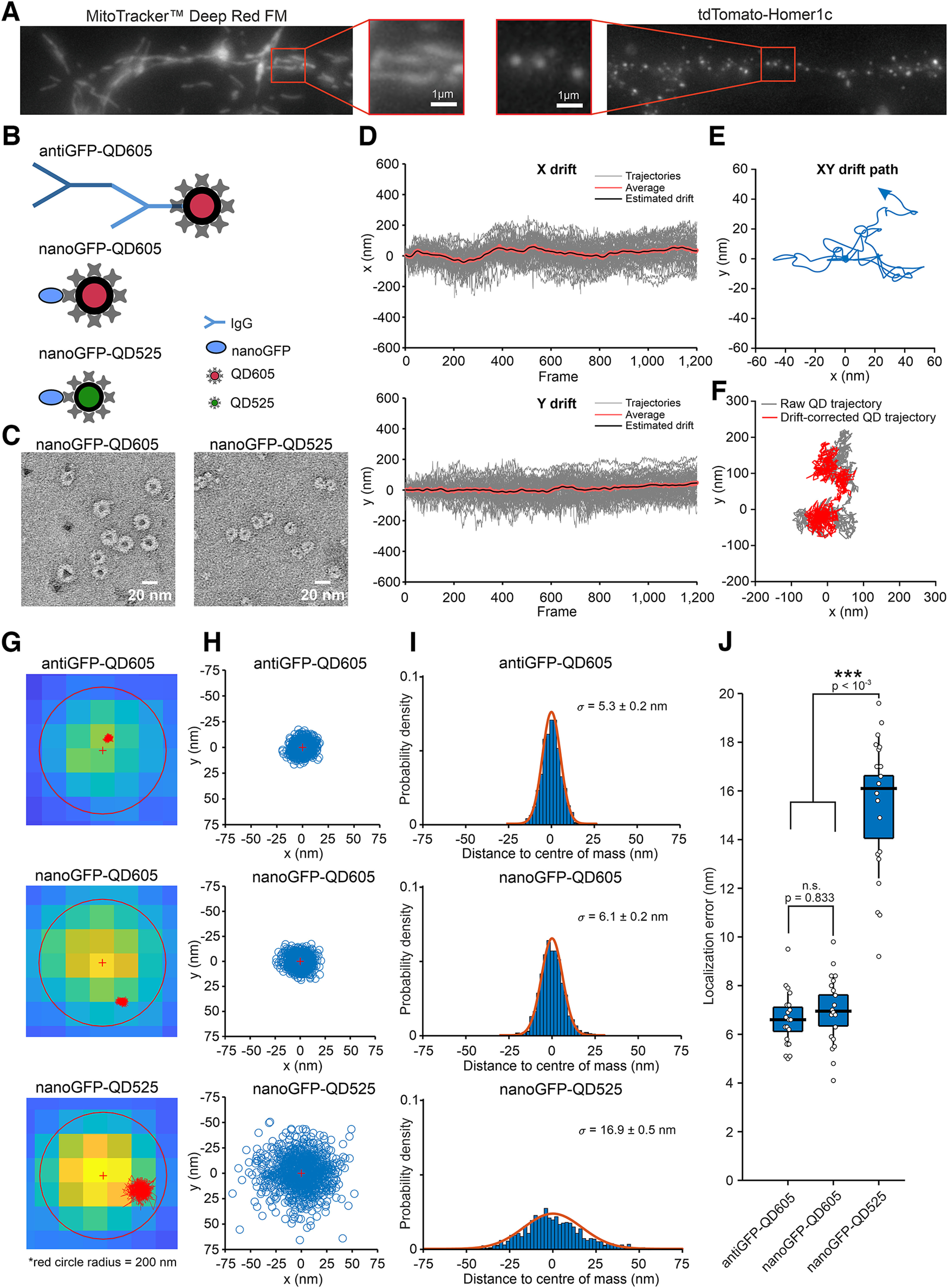
Drift correction and localization error of QD probes in fixed hippocampal neurons expressing the YFP-tagged GluN1-1a subunit. ***A***, Representative images of hippocampal neurons. Left, Neurons were stained by incubation in 20 nm MitoTracker Deep Red FM marker (catalog #M22426, Thermo Fisher Scientific) for 30 s in Neurobasal medium. Right, Neurons were infected with a lentivirus expressing the synaptic protein tdTomato-Homer1c. ***B***, Schematic diagram depicting the three QD-based probes used in this study. The antiGFP-QD605 probe contains the rabbit anti-GFP IgG antibody combined with a secondary IgG antibody conjugated to QD605. The nanoGFP-QD605 probe contains an anti-GFP nanobody conjugated to QD605, while the nanoGFP-QD525 probe contains the anti-GFP nanobody conjugated to QD525. ***C***, Negatively stained samples of both nanoGFP-QD probes were imaged at a magnification of 60,000× and a pixel size of 1.939 Å/px by TEM. Measured average diameters of both nanoGFP-QD probes ± SEM were 16.2 ± 0.4 nm (nanoGFP-QD525) and 20.4 ± 0.6 nm (nanoGFP-QD605; *n* ≥ 30). ***D***, Example of drift estimated from multiple QD trajectories of the YFP-GluN1-1a subunit. ***E***, The corresponding *xy* drift path. ***F***, Example of raw QD trajectories (gray) and the drift-corrected QD trajectories (red) obtained by subtracting the drift path. ***G***, Example images of fixed hippocampal neurons expressing the synaptic marker tdTomato-Homer1c (background pixels) and the YFP-GluN1-1a subunit labeled and tracked with the indicated QD-based probes (red). ***H***, Scatter plots of all QD localizations; the red “+” indicates the mean value in both axes. ***I***, Histograms showing the distribution of the distances between each QD localization shown in ***H***; the data were fitted with a Gaussian function, and the corresponding sigma (σ) values are indicated. ***J***, Box plot summarizing the Gaussian fits performed on fixed QDs (*n* = 20/group); one-way ANOVA *F*_(2,57)_ = 121.84, *p* < 0.0001 followed by Bonferroni's multiple-comparisons test with *p*-values denoted in the figure. The average (mean ± SEM) localization errors were σ = 6.62 ± 0.25 nm (antiGFP-QD605), σ = 6.98 ± 0.32 nm (nanoGFP-QD605), and 15.34 ± 0.66 nm (nanoGFP-QD525).

Drift that occurs during data collection can be a major source of error in the mean square displacement (MSD) analysis (Michalet, 2010). We first attempted to use fluorescent fiducial markers; however, these markers are not spectrally compatible with QDs. Therefore, we developed our own drift correction tool that directly uses the QD signals, given that the wide field of view (82 × 82 µm) typically includes hundreds to thousands of QDs in each image. Because synaptic QD trajectories are spatially extremely limited, and, assuming that their movements are random, we assumed that by averaging a large number of QD trajectories common movements representing drift can be extracted. The drift vector calculated using this approach was subtracted from each QD trajectory (Fig. 1*D–F*), which allowed us to achieve higher localization accuracy (Fig. 1*F*). Drift was always corrected to time “0” (the start of acquisition) to ensure the higher colocalization accuracy with the tdTomato-Homer1c images acquired just before measuring the mobility of the QD-labeled YFP-GluN/GFP-GluN subunits. Any QD trajectories that could not be corrected for drift—typically because of an insufficient number of QD trajectories from the imaged field of view—were excluded from further analysis.

### Mean square displacement

MSD analysis is a technique commonly used to study the motion of colloidal particles, estimate the diffusion characteristics of particles, and/or determine the type of motion (i.e., free diffusion, active transport, or confined diffusion; Michalet, 2010). We used the free MATLAB tool “MSDanalyzer” (Tarantino et al., 2014) to analyze the QD trajectories obtained using TrackMate, which can deal with trajectories of different lengths, time points of onset, and missing detections (a typical characteristic of our tracking data). We used a combination of TrackMate and MSDanalyzer as a validated and published solution because of its ease of implementation in our MATLAB scripts. We used MSDanalyzer to assemble MSD curves and to calculate the instantaneous *D* value for each QD trajectory as the slope obtained from a linear fit of the first 5 points (excluding the zero time delay point) in the MSD curves.

### Sorting into synaptic/extrasynaptic QD trajectories

MSD analysis works only under the assumption that all particles undergo the same type of motion. The movement of receptors in the cell membrane is always spatially constrained and never based purely on free diffusion. The least restricted diffusion occurs at the dendritic shaft surface, as the movements of the receptors are limited only by the relatively large surface area of the dendritic membranes. Synaptic movement, on the other hand, is a highly confined type of movement limited to a few hundred nanometers. Inclusion of several types of motion in MSD analysis could result in increased inaccuracy of the obtained characteristics; therefore, it is important to accurately distinguish between synaptic and extrasynaptic QD trajectories of NMDARs to obtain a good estimate of the diffusion characteristics.

### Determination of synaptic regions

**Figure 2.**
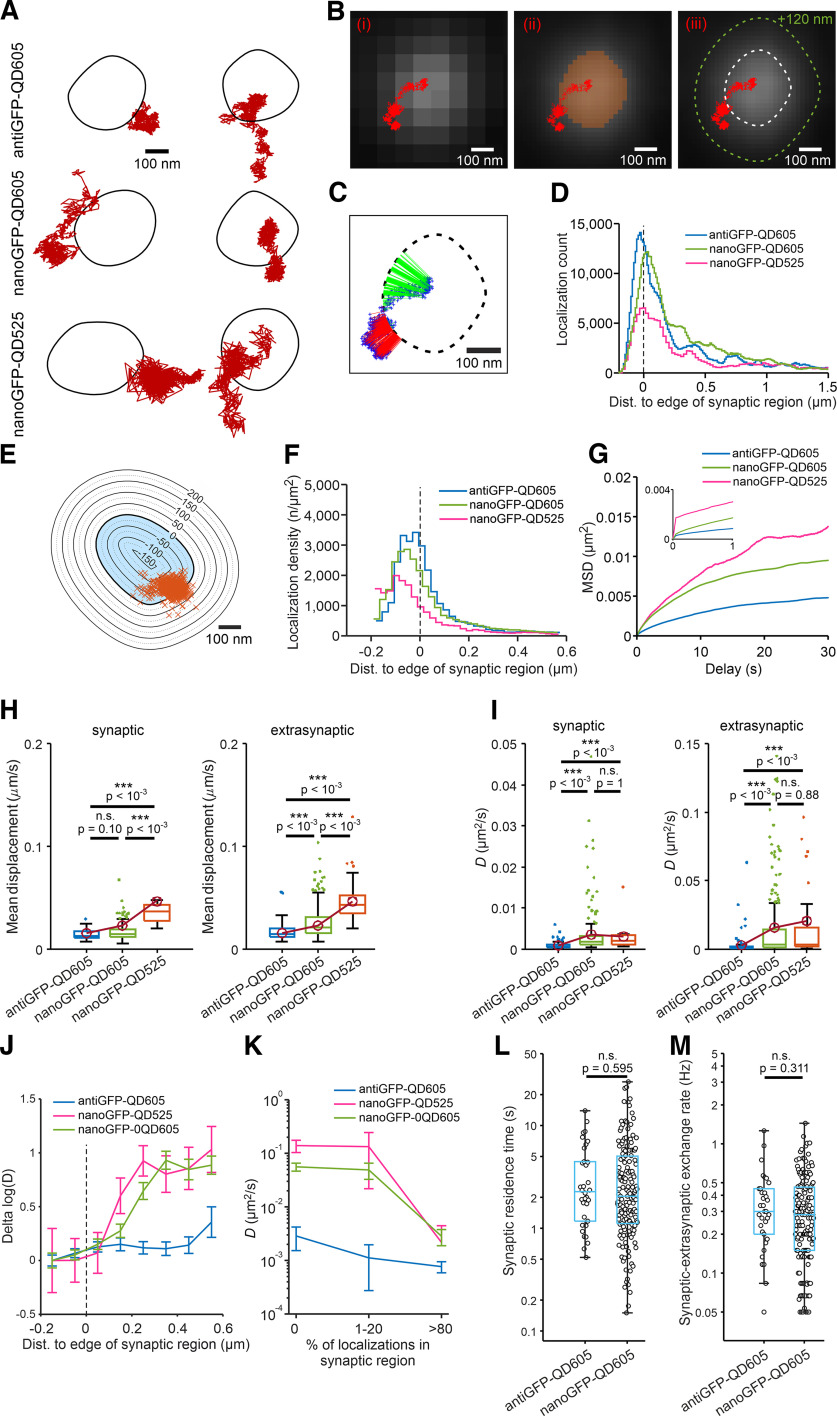
Evaluating the tracking performance of three QD-based probes in cultured hippocampal neurons expressing YFP-GluN1-1a subunit. ***A***, Examples of surface QD trajectories (red) of individual NMDARs measured in hippocampal neurons coexpressing YFP-GluN1-1a subunit and the synaptic marker tdTomato-Homer1c to define the synaptic region (indicated by the black boundaries). The neurons were labeled with the indicated QD-based probes and imaged for 60 s with a 50 ms interval between frames. ***Bi–iii***, Definition of the synaptic region using the tdTomato-Homer1c signal. The pixelized tdTomato-Homer1c signal (***i***) was interpolated (***ii***), and local gradient-based thresholding (see Materials and Methods) was used to define the synaptic region with subpixel precision (***iii***, dashed white line), and to define extended synaptic region (***iii***, dashed green line). ***C***, Absolute distance of QDs to the edge of synaptic region was measured for each trajectory time point. ***D***, Histogram of the absolute distances between each QD localization and the edge of the synaptic region (indicated by the vertical dashed line at 0); *n* > 132,226 QD localizations per probe. Negative and positive distances indicate QD localizations inside and outside of the synaptic region, respectively. ***E***, The synaptic region was extended by concentric zones with step of 25 nm, a localization density of QD probes was measured as the number of localizations in the concentric zone divided by the area of the zone. ***F***, Histogram of localization density plotted against distance to the edge of the synaptic region (indicated by the vertical dashed line at 0); *n* > 220 synaptic regions per probe. Negative and positive distances indicate QD localizations inside and outside of the synaptic region, respectively. ***G***, MSD curves for synaptic QD trajectories plotted against time delay; the curves were vertically aligned by subtracting the value with a “0” s delay; the inset shows the initial part of curves without subtraction. ***H***, Box plots summarizing the mean frame-to-frame displacement of NMDARs labeled with the indicated QD probe sorted into synaptic and extrasynaptic pools defined using Method 1. Note that mean displacement is the product of the actual displacement of the QD probe and the localization error; K–W test: *F*_(2,280)_ = 75.8736, *p* < 10^−4^ (for synaptic) and *F*_(2,395)_ = 82.187, *p* < 10^−4^ (for extrasynaptic); median values [synaptic, extrasynaptic] (µm/s): antiGFP-QD605 [0.0132, 0.0152]; nanoGFP-QD605 [0.0146, 0.0209]; nanoGFP-QD525 [0.0371, 0.0429]. ***I***, Box plots summarizing the *D* values derived by the linear fit of the MSD curves (see Materials and Methods); K–W test: *F*_(2,280)_ = 36.0756, *p* < 10^−4^ (for synaptic) and *F*_(2,395)_ = 90.9383, *p* < 10^−4^ (for extrasynaptic); median *D* values [synaptic, extrasynaptic] (µm^2^/s): antiGFP-QD605 [0.0007, 0.0010]; nanoGFP-QD605 [0.0021, 0.0037]; nanoGFP-QD525 [0.0019, 0.0038]. ***J***, Change in *D* value plotted against the distance from the edge of the synaptic region (indicated by the vertical dashed line). QD trajectories were binned based on the distance from the center of the QD trajectory to the edge of the nearest synaptic region, in 100 nm bins. The error bars represent the 95% confidence interval. ***K***, Mean *D* values for the QD trajectories plotted against the percentage of time the QD trajectories spent inside the synaptic region; note that the *y*-axis is logarithmic, and error bars represent the 95% confidence intervals. ***L***, ***M***, Box plots summarizing the synaptic residence time (i.e., the mean time spent in the synaptic region) in seconds (***L***); median values: 2.27 s (antiGFP-QD605) and 2.05 s (nanoGFP-QD605); and the synaptic–extrasynaptic exchange rate (i.e., the number of transitions between the synaptic and extrasynaptic regions, in Hz (***M***); median values: 0.30 Hz (antiGFP-QD605) and 0.28 Hz (nanoGFP-QD605). Box plots in both ***L*** and ***M*** show log-transformed data with labels on the vertical axis kept as nontransformed reading times in seconds and frequencies in Hz. Data passed a D'Agostino–Pearson's normality test after log-transformation; Student's *t* test: *t*_(187)_ = 0.53211 (residency time); *t*_(187)_ = 1.0155 (exchange rate), with *p*-values denoted in plots.

To obtain structural information, we obtained a diffraction-limited microscopy *z*-stack of the YFP/GFP and tdTomato signals around the focal plane in which we subsequently measured the mobility of the QD probes. This allowed us to determine the centers and edges of the fluorescent tdTomato-Homer1c signals with subpixel precision. We first located individual synapses by detecting local maxima; the exact position of the center of each synapse was then calculated by finding the center of symmetry around the local maxima. We determined the signal edges using local intensity gradient-based thresholding in MATLAB. The process of defining the synaptic region is shown in Figure 2*Bi–iii*. The raw image (Fig. 2*Bi*) was first oversampled (i.e., upscaled) by a factor of four to achieve fine image interpolation. Subsequently, we designated all adjacent pixels with brightness >50% of the brightness maximum of/in the center of the synapse as the synaptic region (Fig. 2*Bii*). The pixelated mask was then contoured using the *bwtraceboundary* function and subsequently converted to a polygon using the *drawpolygon* function. The result was an accurate contour defining the synaptic region (Fig. 2*Biii*). Where needed, the contour was then expanded using the *polybuffer* function, which ensured a uniform expansion in all directions.

We used three methods to define the synaptic region and then compared the results using our experimental data, thereby avoiding the potential influence of artificial boundaries.

#### Method 1.

**Figure 3.**
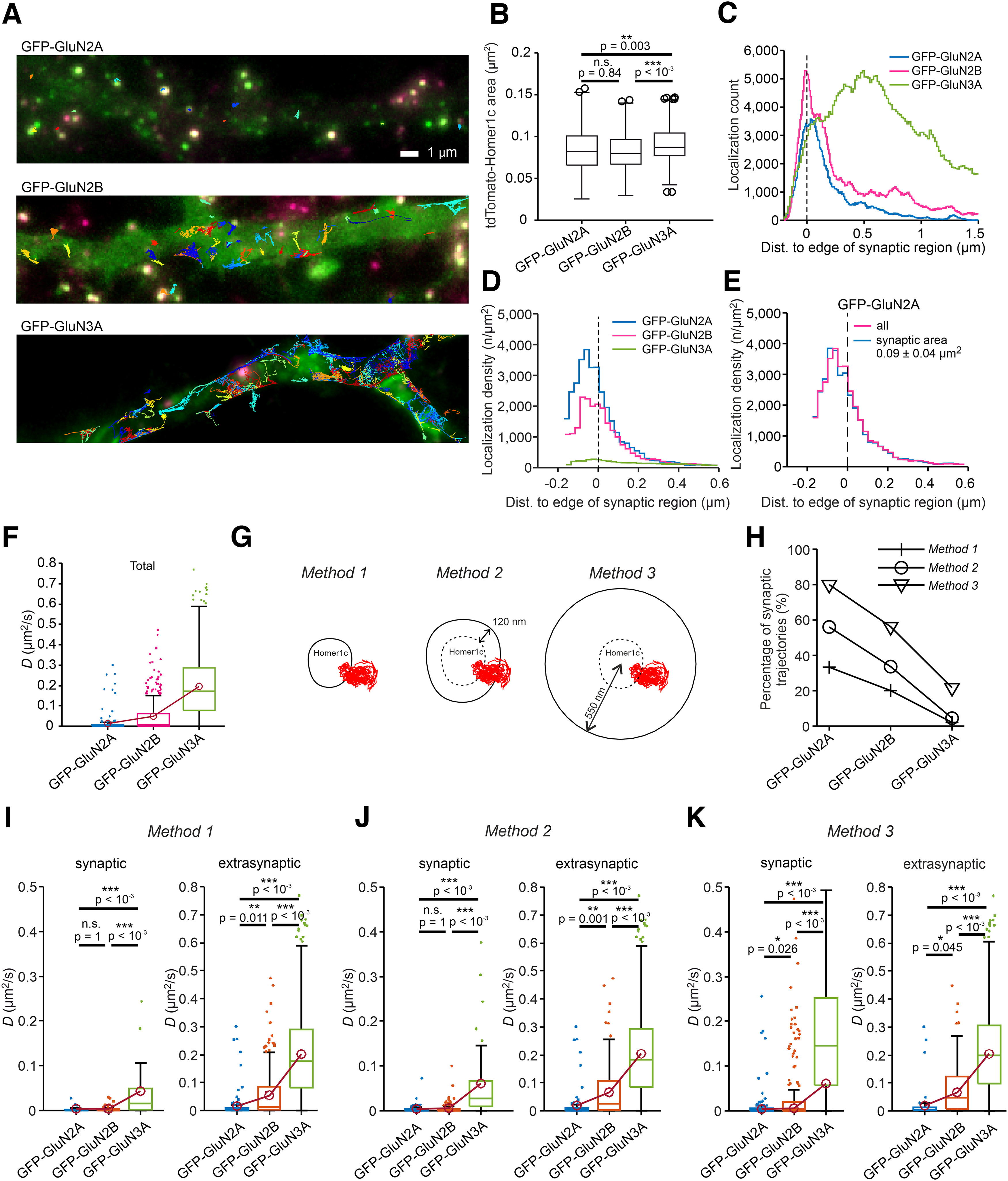
Surface mobility of NMDARs measured using the nanoGFP-QD605 probe in hippocampal neurons expressing GFP-GluN2A, GFP-GluN2B, or GFP-GluN3A subunits. ***A***, Representative surface QD trajectories of GFP-GluN2A-containing, GFP-GluN2B-containing, or GFP-GluN3A-containing NMDARs (green) in hippocampal neurons expressing tdTomato-Homer1c (red) tracked using the indicated nanoGFP-QD605 probes (colored trajectories). ***B***, Box plot summarizing the area of the synaptic region based on the tdTomato-Homer1c signal using Method 1; one-way ANOVA: *p* < 10^−4^, *F*_(2,1526)_ = 22.6 followed by Bonferroni's multiple-comparison test with *p*-values indicated in the plot. ***C***, Histogram of the absolute distance between each QD localization and the edge of the nearest synaptic region (indicated by the vertical dashed line at 0) for NMDARs containing the indicated GFP-GluN subunits; Kolmogorov–Smirnov test: *p* = 0.152 (GFP-GluN2A vs GFP-GluN2B); *p* < 10^−4^ (GFP-GluN2A vs GFP-GluN3A); *p* = 0.004 (GFP-GluN2B vs GFP-GluN3A; *n* > 155,909 QD localizations/group). ***D***, The nanoGFP-QD605 localization density for NMDARs containing the indicated GFP-GluN subunits plotted against distance to the edge of the synaptic region (indicated by the vertical dashed line at 0). ***E***, Localization density of all nanoGFP-QD605-labeled GFP-GluN2A-containing NMDARs compared with the localization density of the GFP-GluN2A-containing NMDARs from synaptic regions limited to a range of 0.092 ± 0.041 µm^2^ (mean ± 2 × SDs; based on data with GFP-GluN3A subunit shown in ***B***). ***F***, Box plot summarizing the *D* values calculated from all (both synaptic and extrasynaptic) QD trajectories; median *D* values: 0.0020 µm^2^/s (GFP-GluN2A); 0.0692 µm^2^/s (GFP-GluN2B); 0.1730 µm^2^/s (GFP-GluN3A). ***G***, Schematic diagrams depicting the three methods used to define the synaptic region (for details, see Materials and Methods). ***H***, Summary of the percentage of synaptic QD trajectories defined as >50% in the synaptic region using the indicated methods. ***I***–***K***, Bottom, Box plots summarizing the *D* values of QD trajectories measured for the synaptic and extrasynaptic pools of NMDARs containing the indicated GFP-GluN subunits, with the synaptic region defined using the indicated method; K–W test: *p* < 10^−4^ (***I–K***, all conditions) followed by Bonferroni's multiple-comparisons test with *p*-values indicated in the plots; median *D* values [synaptic, extrasynaptic] (µm^2^/s): ***I*** (Method 1): GFP-GluN2A [0.0011, 0.0025]; GFP-GluN2B [0.0012, 0.0125]; GFP-GluN3A [0.015, 0.1766]; ***J*** (Method 2): GFP-GluN2A [0.0015, 0.0039]; GFP-GluN2B [0.0013, 0.0234]; GFP-GluN3A [0.0216, 0.182]; ***K*** (Method 3): GFP-GluN2A [0.0016, 0.0088]; GFP-GluN2B [0.0028, 0.0462]; GFP-GluN3A [0.1168, 0.1964].

The synaptic region was defined based on the tdTomato-Homer1c signal (see above). QD trajectories were designated as synaptic if >50% of their localizations were within the defined synaptic region; otherwise, their trajectories were designated as extrasynaptic (Fig. 3*G*).

#### Method 2.

The synaptic region was defined as a perimeter of 120 nm (“perisynaptic” area) from the edge of the area (Pérez-Otaño et al., 2006) delineated by the tdTomato-Homer1c signal using Method 1 (Fig. 3*G*). QD trajectories with >50% localizations in the defined synaptic region were designated as synaptic; otherwise; their trajectories were designated as extrasynaptic.

#### Method 3.

The distance of each QD trajectory point from the center of the nearest synapse was measured, and the average distance of each QD trajectory from the nearest synapse was calculated and used to automatically reclassify each QD trajectory as synaptic or extrasynaptic. QD trajectories whose center was <550 nm from the center of the synapse were designated as synaptic; otherwise, their trajectories were designated as extrasynaptic (Fig. 3*G*). This approach was used previously to study the mobility of AMPA receptors (AMPARs; Lee et al., 2017).

### Analysis of the probe localization relative to the edge of the synaptic region

To refine the analysis beyond the binary classification into synaptic and extrasynaptic populations of NMDARs, we measured the distance between the position of each localized QD and the edge of the nearest synaptic region, and plotted a histogram showing the localization count versus this distance (Fig. 2*C*,*D*). In addition, we defined concentric zones within and around the synaptic region with a 25 nm step and then calculated the localization density as the number of localizations observed within the concentric zone divided by the area of the zone (Fig. 2*E*,*F*). As the areas of the equidistantly spaced zones are not equal (the more central zones are smaller; Fig. 2*E*), the localization density profile in Figure 2*F* differs from the unnormalized count histogram in Figure 2*D*. The localization density shown in Figure 2*F* provides a proper estimate of the local surface density for probes in the synaptic and perisynaptic regions. At larger distances outside of the synapse, however, the concentric zones start including regions that are outside of the dendrite and do not contain any membrane; the zone area then does not accurately quantify the membrane surface from which the QD localizations were gathered. At these larger distances, the unnormalized count in Figure 2*D* provides a more relevant spatial profile of probe density. Similarly, to evaluate the probe mobility within the assembled QD trajectories without the influence of binary sorting, we grouped the trajectories based on the binned distance from the center of the QD trajectory to the edge of the nearest synaptic region, and computed the mean diffusion coefficient for each 100 nm bin (Fig. 2*J*). In Figure 2*K*, we report the mean diffusion coefficients for QD trajectories that were grouped according to the indicated percentage thresholds used to classify QD trajectories as synaptic.

### Universal point accumulation imaging in nanoscale topography

Hippocampal neurons cultured in glass-bottom dishes were washed and placed in IS equilibrated at 37°C. Universal point accumulation imaging in nanoscale topography (uPAINT) experiments were performed using a Zeiss Elyra 7 microscope [63×/1.46 oil-objective with correction collar used to optimize PSF; pco.edge sCMOS camera, pco (frame rate, 50 ms; exposure time, 45 ms)] under HiLo [epi-total internal reflection fluorescence (TIRF)] illumination at 37°C. Specifically, after a region of interest (ROI) selection, a *z*-stack image of the tdTomato-Homer1c signal was first acquired. Then, the nanoGFP-ATTO647N probe was added into IS and diluted to a final concentration of ∼0.5 nm to sparsely and stochastically label the YFP-GluN/GFP-GluN subunits at the cell surface. The signals of the nanoGFP-ATTO647N probe were excited with a 642 nm laser, and the emission was detected using an LP 655 filter. A whole-microscope heated enclosure along with real-time *z*-drift piezoelectric correction by the Definite Focus microscope (Zeiss) ensured temperature stability and no detectable drift over a 60 s time span. The typical duration of uPAINT trajectories was within ∼10 s, and we had to take approximately five times more images than with QD-based probes to obtain a comparable number of localizations. To minimize any possible effect of drift on the registration of QD localizations with respect to the tdTomato-Homer1c signal, we divided the uPAINT imaging into five blocks, where one image was taken each time with the tdTomato-Homer1c signal immediately followed by 60-s-long imaging with nanoGFP-ATTO647N trajectories. All uPAINT experiments were acquired within 20 min after addition of the nanoGFP-ATTO647N probe.

After the acquisition, the analysis procedure was identical to that of the QD probes (i.e., the positions of the ATTO647N signals were located in each frame and assembled into trajectories using the TrackMate plugin integrated into our own MATLAB-based software). The TrackMate parameters were chosen empirically to achieve the best tracking performance, as follows: subpixel localization = true; radius = 0.35 µm; threshold = 0.9; median filter = true; spot quality threshold = 12; and track assembly parameters: linkage maximum distance = 0.2 µm; gap closure maximum distance = 0.3 µm; maximum interframe gap = 10 frames. Synaptic localizations were determined based on their colocalization with synaptic regions defined by Method 1.

### Direct stochastic optical reconstruction microscopy

Testing of the optimal concentration of the nanoGFP-AF647 probe was performed on hippocampal neurons cultured on glass coverslips. Briefly, neurons were washed with Neurobasal medium and then immediately incubated for 7 min with nanoGFP-AF647 probe diluted from a stock solution of ∼1 mg/ml in Neurobasal medium, followed by four rapid washing steps (2× Neurobasal medium, 2× PBS). Samples were then fixed with 4% PFA containing 4% sucrose in PBS for 7 min, permeabilized with 0.25% Triton X-100 in PBS for 5 min, and labeled with primary rabbit anti-GFP antibody (1:1000; Merck) followed by goat anti-rabbit antibody conjugated with AF488 (1:1000; Thermo Fisher Scientific). Cells were then mounted with ProLong Antifade reagent (Thermo Fisher Scientific). Images were acquired using a confocal microscope (model FV10i, Olympus) equipped with a 60×/1.35 oil-immersion objective and a dual-channel photomultiplier (512 × 512 pixels; dwell time, 3.8 μs/pixel; detector voltage set to unsaturated range; Kolcheva et al., 2023). All parameters were the same when each dataset was acquired. At least four separate 10-μm-long segments of secondary or tertiary dendrites per neuron were analyzed using ImageJ software as described previously (Skrenkova et al., 2020).

For direct stochastic optical reconstruction microscopy (dSTORM) staining, infected hippocampal neurons cultured in glass-bottom dishes were washed once with Neurobasal medium and then immediately incubated for 7 min with a saturating concentration of nanoGFP-AF647 probe diluted in Neurobasal medium. This was followed by four rapid washing steps (2× Neurobasal medium, 2× PBS) and fixation with 4% PFA containing 4% sucrose in PBS for 7 min. The fixative was then washed thoroughly, and the samples were stored in PBS at 4°C. The dishes were then filled with freshly prepared imaging buffer (IB) containing 50 mm Tris, pH 8.0, 10 mm NaCl, 50 mm mercaptoethylamine, 8% (w/v) glucose, 0.5 mg/ml glucose oxidase, 40 µg/ml catalase, and 2 mm cyclooctatetraene (all from Merck). The dishes with IB were then covered with a coverslip and black tape to maintain an oxygen-free environment. dSTORM imaging was performed on the N-STORM module of a Nikon Ti-E microscope; before each experiment, the correction ring on the HP Apo 100× oil-immersion objective, NA 1.49, was adjusted for an optimal point scatter function using TetraSpec microspheres (0.1 µm; Thermo Fisher Scientific). When an ROI was found, a wide-angle *z*-stack image of the tdTomato-Homer1c signal was first taken. The AF647 signal was excited by a 647 nm laser set to 100% laser power (∼1000 W/cm^2^) and a 405 nm laser set to 2% laser power (∼5 W/cm^2^) under epi-TIRF illumination and was detected on an Andor iXon Ultra DU897 EM CCD camera (resolution, 512 × 512; pixel size, 160 nm; EM gain, 300). For each ROI, 30,000 frames were collected at a rate of 30 frames/s.

The individual localizations were obtained with the Fiji plugin ThunderSTORM (Ovesný et al., 2014). The threshold for localization identification was set to five times the image background, and peaks were fitted with the Gaussian function. The first 500 images were filtered out. The drift was corrected using the software cross-correlation algorithm (bins = 14; magnification = 5; smoothing factor = 0.25). Before colocalizing tdTomato-Homer1c and dSTORM images, TetraSpec microspheres were used for chromatic aberration corrections in MATLAB. MATLAB was also used for further analysis. Synaptic localizations were determined based on their colocalization with the synaptic region defined by Method 1.

### Electrophysiology

Whole-cell patch-clamp recordings of HEK293 cells were performed 24–48 h after transfection using an Axopatch 200B amplifier (Molecular Devices). Borosilicate glass pipettes (tip resistance, 3–6 MΩ) were prepared using a P-97 micropipette puller (Sutter Instrument) and filled with a solution containing the following (in mm): 125 gluconic acid, 15 CsCl, 5 BAPTA, 10 HEPES, 3 MgCl_2_, 0.5 CaCl_2_, and 2 ATP-Mg salts, with pH adjusted to 7.2 with CsOH. The extracellular recording solution (ECS) contained the following (in mm): 160 NaCl, 2.5 KCl, 10 HEPES, 10 glucose, 0.2 EDTA, and 0.7 CaCl_2_, with pH adjusted to 7.3 using NaOH. ECS containing the agonists l-glutamate and/or glycine were applied to the cells using a microprocessor-controlled rapid perfusion system with a solution exchange rate of ∼20 ms. Concentration–response curves for the effect of agonists were obtained by fitting the electrophysiological data with the following equation:

$$I=I_{\text{max}}/\left(\text{1 }+{\left({\text{EC}}_{\text{5}0}/\left[\text{agonist}\right]\right)}^{h}\right),$$

where *I* is the current amplitude at the given agonist concentration, *I*_max_ is the maximum peak current amplitude in response to the agonist, EC_50_ is the agonist concentration that elicited the half-maximal response, [agonist] is agonist concentration (in μm), and *h* is the apparent Hill coefficient. Time constants of desensitization (τ_des_) were obtained for GluN1/GluN3A receptors in response to the indicated concentrations of glycine using the following equation:

$$\tau_{\text{des}}=\left(\tau_{\text{fast}}*{\text{A}}_{\text{fast}}+\tau_{\text{slow}}*{\text{A}}_{\text{slow}}\right)/\left({\text{A}}_{\text{fast}}+{\text{ A}}_{\text{slow}}\right),$$

where τ_des_ is the weighted time constant of desensitization, τ_fast_ and A_fast_ are desensitization time constant and amplitude, respectively, of the fast component, and τ_slow_ and A_slow_ are the desensitization time constant and amplitude, respectively, of the slow component. To obtain electrophysiological recordings of GluN3A-containing NMDARs from hippocampal neurons, we used IS containing 1 μm tetrodotoxin (to inhibit synaptic activity), 10 μm bicuculline (to inhibit GABA receptors), 10 μm strychnine (to inhibit glycine receptors), and 50 μm d-2-amino-5-phosphonovalerate (d-APV; to inhibit GluN1/GluN2 receptors). Current responses were induced by 100 μm glycine or 100 μm glycine in combination with 0.5 μm CGP-78608. To verify the KO efficiency of GluN2A and GluN2B subunits in mouse neurons, current responses were induced by 100 μm NMDA in the continuous presence of 20 μm glycine; Mg^2+^ and d-APV were omitted. All electrophysiological recordings were performed at room temperature at a membrane potential of −60 mV. The electrophysiological data are presented as the mean ± SEM, and differences between groups were analyzed using the Student's *t* test (SigmaStat 3.5, Systat Software).

### Experimental design and statistical analyses

Where possible, and unless indicated otherwise, summary data are presented using a combined box plot including all data points with boxes denoting median value (horizontal line) and SD (the box); if the dataset contained too many data points, the data are presented using a box plot (MATLAB *boxchart*) showing the lower and upper quartile (the box), median value (the horizontal line), minimum/maximum values (the whiskers), and outlying data points (circles). The *D* values varied widely; therefore, we either plotted these values on a logarithmic scale or sorted them into two pools (synaptic and extrasynaptic) and plotted them on a linear scale. In the text, summary data are presented as the mean ± SEM, the median, or the mean ± SD. All statistical analyses were performed using MATLAB 2022b. Group differences were analyzed using either Student's *t* test or a one-way ANOVA (*anova1* function) along with the D'Agostino–Pearson normality test. Differences between data that did not unconditionally satisfy the normality condition were analyzed using a nonparametric version of the one-way ANOVA, the Kruskal–Wallis (K–W) test (*kruskawallis* function). In cases in which the null hypothesis was rejected, we performed a multiple pairwise comparison (*multicompare* function) with a conservative Bonferroni procedure (typically for the *D* values) to subsequently analyze the difference between groups. For non-normally distributed data, we corrected for skewed distributions using a logarithmic transformation to stabilize the variance around the mean (typically for the synaptic residence time and synaptic–extrasynaptic exchange rate) and then performed a one-way ANOVA (*anova1* function). Differences with a *p*-value < 0.05 were considered significant, and significance is indicated in the figures as follows: *p < 0.050, **p < 0.010, ***p < 0.001, and n.s., not significant. The Kolmogorov–Smirnov test (*kstest* function) was used for quantification of a distance between two empirical distributions. All analytical tools used in this study will be made available to readers on request.

## Results

### Analysis of the performance of three QD-based probes for tracking surface NMDARs in cultured neurons

Our first step was to determine the most appropriate QD-based probe, establish the optimal experimental conditions, and then develop analytical tools to comprehensively analyze the surface trajectories of specific probe-labeled GluN subunits in cultured hippocampal neurons, including the most accurate method for differentiating between synaptic and extrasynaptic regions. For this study, we used cultured hippocampal neurons, as these neurons are the most commonly used cell type for studying the mobility of both AMPARs and NMDARs (Dupuis and Groc, 2020; Groc and Choquet, 2020); and only mature neurons with a pyramidal cell body shape at DIV14 to DIV16 were used for our measurements. We initially considered using MitoTracker Deep Red FM as a marker for labeling excitatory synapses, similar to previous studies (Tardin et al., 2003; Groc et al., 2004; Heine et al., 2008); however, our experiments using short-term staining of hippocampal neurons did not provide a clear pattern of synaptic labeling (Fig. 1*A*). Given that overexpressing the neuronal protein Homer1c does not alter synaptic transmission (Hennou et al., 2003) compared with overexpressing PSD-95 (Béïque and Andrade, 2003), and given that a fluorescent protein-tagged Homer1c was used previously as a synaptic marker to study the surface mobility of AMPARs (Lee et al., 2017), we used a commercially available lentiviral construct expressing tdTomato-Homer1c; when expressed in cultured hippocampal neurons, this protein had a typical synaptic localization pattern (Fig. 1*A*).

Next, we attempted to identify the ideal fluorescent probe for detecting the surface mobility of NMDARs. Our ultimate goal was to compare surface mobility between various NMDAR subtypes using a single fluorescent probe. We previously showed that several YFP-/GFP-tagged GluN subunits, such as YFP-GluN1-1a, GFP-GluN2A, GFP-GluN2B, and GFP-GluN3A, can be expressed in hippocampal neurons (Kaniakova et al., 2012; Skrenkova et al., 2019). We therefore focused on creating GFP-based probes. The ideal fluorescent probe for our experiments should be as small as possible to enter the synaptic cleft (Zuber et al., 2005; Pósfai et al., 2016), and it should be suitable for detecting the trajectories of labeled GluN subunits with high resolution and minimal photobleaching during our desired imaging duration of ∼60 s. We therefore chose an anti-GFP nanobody (nanoGFP) as the protein component of our probes because of its small size and strong specificity for binding GFP (Kubala et al., 2010); we also used our previously published antiGFP-QD605 probe for comparison (Skrenkova et al., 2018).

We opted to conjugate the nanoGFP to both QD605 (resulting in the nanoGFP-QD605 probe) for direct comparison with the antiGFP-QD605 probe, as well as with the smallest commercially available QD, QD525 (resulting in the nanoGFP-QD525 probe; Fig. 1*B*). We first measured the nanoGFP probes using FCS and found that the nanoGFP-QD525 and nanoGFP-QD605 probes have hydrodynamic radii of ∼9 and ∼12 nm, respectively, consistent with the expected size difference between the two QD cores (Table 1). In a previous study of NMDAR mobility, the authors included 1% (w/v) BSA to block nonspecific protein-binding sites on the cells surface (Ferreira et al., 2017). However, because BSA can increase the size of QDs (Le et al., 2020), BSA was replaced with 1.25% (w/v) casein in a recent study of AMPAR surface mobility (Lee et al., 2017). To determine whether to use BSA or casein in our subsequent experiments, we used FCS to measure the hydrodynamic radii of both nanoGFP-QD probes in the presence of 1% BSA and 1.25% casein and found that BSA had a larger effect on increasing the hydrodynamic radii of the probes compared with casein (Table 1); thus, except where stated otherwise, we used 1.25% casein to block nonspecific protein-binding sites at the cell surface. We did not use FCS to measure the antiGFP-QD605 probe based on the need for prohibitively large amounts of both the primary and secondary antibodies. When imaged in negative stain TEM experiments, the diameter of the nanoGFP-QD525 probe (∼16 nm) was smaller than that of the nanoGFP-QD605 probe (∼20 nm); moreover, both nanoGFP-QD probes were relatively homogeneous and monodisperse (Fig. 1*C*). The limitations of the negative stain TEM method preclude an assessment of the size of the protein parts of the QD probes. However, the known molecular weight of the protein part of the antiGFP-QD605 probe [i.e., one molecule of primary IgG, ∼150 kDa; one molecule of secondary F(ab')2, ∼100 kDa] is markedly larger than one molecule of nanoGFP (∼15 kDa; see Introduction). This indicates a smaller size of the nanoGFP-QD605 probe and suggests that it is more optimal for studying the surface mobility of NMDARs compared with the antiGFP-QD605 probe.

**Table 1.** Summary of the hydrodynamic radii of the nanoGFP probes measured using FCS

Probe	Buffer	*R*_H_ (nm)
nanoGFP-QD525	IS	8.8 ± 0.4
nanoGFP-QD525	IS + BSA	11.1 ± 0.6
nanoGFP-QD525	IS + casein	9.1 ± 0.5
nanoGFP-QD605	IS	12.0 ± 0.5
nanoGFP-QD605	IS + BSA	12.9 ± 0.5
nanoGFP-QD605	IS + casein	12.4 ± 0.5

The *R*_H_ of the indicated nanoGFP-QD probes was calculated using the Stokes–Einstein equation (for details, see Materials and Methods). Data are the mean ± SEM.

As we observed that lateral drift generally occurred during our imaging, we always corrected the microscopy data for drift using our drift correction tool (Fig. 1*D–F*; see also Materials and Methods). Next, we aimed to experimentally measure the localization accuracy of all three QD-based probes in our experimental setup. Therefore, we fixed QD probes in hippocampal neurons expressing the YFP-GluN1-1a subunit and then imaged them for 60 s as described in the Materials and Methods (Fig. 1*G*). We then measured the localization accuracy of all three QD-based probes by fitting a Gaussian function to all localizations and expressed the localization error as the SD around the average position determined over the 60 s imaging time (Fig. 1*H*,*I*). We found that the two QD605-based probes (i.e., antiGFP-QD605 and nanoGFP-QD605) had a localization accuracy of ∼6–8 nm (Fig. 1*I*,*J*); in contrast, the localization accuracy of the nanoGFP-QD525 probe was ∼14–17 nm (Fig. 1*I*,*J*). This difference between the QD605 and QD525 probes is in part because of spectral overlap between QD525 and GFP emission (despite QD525 and GFP having different excitation wavelengths), and mostly because of the fact that the light emitted by QD525 is considerably less bright compared with QD605.

To determine the efficiency with which individual QD probes can monitor the surface (including synaptic) mobility of NMDARs, we imaged the QD525 and QD605 signals in cultured hippocampal neurons expressing both YFP-GluN1-1a subunit and tdTomato-Homer1c (Fig. 2*A*). Local thresholding and contouring of the tdTomato-Homer1c signal were used to identify the synaptic regions with subpixel precision (see Method 1 for identification of the synaptic regions; Fig. 2*B*). We first analyzed the distribution of distances between the QD trajectory points and the edge of the synaptic region (Fig. 2*C*); the localization count was plotted in a histogram versus distance. This showed that all three QD-based probes had the highest count in the vicinity of the synaptic edge (Fig. 2*D*). Second, we analyzed the spatial profile of the surface density of the probes. To determine the surface density, we defined equally spaced concentric zones within and outside each synaptic region with a 25 nm step (Fig. 2*E*), and then calculated the localization density as the number of localizations observed within the concentric zone divided by the area of the zone. The localization densities averaged from all synaptic regions were plotted against the distance from the edge of the synaptic region (Fig. 2*F*). This analysis showed that all probes had peak densities within the synaptic region between −100 and −50 nm, with density peaks shifting slightly toward the center of the synaptic region in order from the presumed largest probe (antiGFP-QD605) to the smallest probe (nanoGFP-QD525). The defined synaptic region was then used to classify QD trajectories as synaptic (defined as QDs that spent >50% of the time in the synaptic region) or extrasynaptic (defined as QDs that spent >50% of the time in the extrasynaptic region).

To evaluate the surface mobility of the QD-based probes, we first calculated the mean “frame-to-frame” displacement between two consecutive frames (Fig. 2*H*). Note that this measurement of mobility is essentially the sum of the actual movement of the QD particle and the localization error of the particle. As expected, the nanoGFP-QD525 probe—which had a larger localization error (Fig. 1*J*)—had higher rates of displacement for both the synaptic and extrasynaptic QD trajectories compared with the antiGFP-QD605 and nanoGFP-QD605 probes (Fig. 2*H*). To avoid the effect of localization error, we conducted standard mean square displacement analysis (Michalet, 2010); this approach calculates the short-time *D* value using the linear fit of the first 5 points in the MSD versus time delay curve. Our analysis showed that the *D* values of both the synaptic and extrasynaptic QD trajectories were lower for the antiGFP-QD605 probe than for both nanoGFP probes, with no significant difference between the nanoGFP-QD525 and nanoGFP-QD605 probes (Fig. 2*I*). In all cases, the *D* values obtained for each QD probe were higher for extrasynaptic QD trajectories compared with synaptic QD trajectories, consistent with previous reports (Groc et al., 2006). Above, we classified QD trajectories into synaptic and extrasynaptic pools by applying a commonly used method based on a sharp threshold for the synaptic region (>50%). To avoid the effect of a rigid threshold set by the defined synaptic region, we also plotted the change in *D* values against the distance from the edge of the synaptic region in 100 nm increments (Fig. 2*J*). We found that both the nanoGFP-QD525 and nanoGFP-QD605 probes had a steep increase in *D* values at increasing distances from the synaptic edge starting ∼100 nm from the synaptic edge. In contrast, the *D* values measured for the antiGFP-QD605 probe were mostly constant at increasing distances. Next, we sorted the QD trajectories into smaller pools based on the percentage of time (intervals: 0%; >0–20%; and >80%) that they spent in the synaptic region (defined by Method 1) and compared the mean *D* values. This approach revealed that the *D* values were smaller for the antiGFP-QD605 probe when compared with both nanoGFP-QD probes, even in the case of >80% intervals of localization in the synaptic region (Fig. 2*K*).

Both NMDARs and AMPARs can move between synaptic and extrasynaptic regions by lateral diffusion (Dupuis and Groc, 2020; Groc and Choquet, 2020). We therefore attempted to determine which QD605-based probe (i.e., antiGFP-QD605 or nanoGFP-QD605; we excluded the nanoGFP-QD525 probe from this experiment because of its relatively high localization error) is better suited for detecting the movement of NMDARs between the synaptic and extrasynaptic regions. We then calculated the synaptic residence time (in seconds) and synaptic–extrasynaptic exchange rate (in hertz). For our analysis, we selected all QD trajectories that had at least four transitions between the synaptic and extrasynaptic regions, and we excluded any transitions lasting ≤100 ms (i.e., fewer than three consecutive frames). Our analysis revealed no significant difference in either synaptic residence time or synaptic–extrasynaptic exchange rate between the antiGFP-QD605 and nanoGFP-QD605 probes (Fig. 2*L*,*M*). To understand how this is compatible with our finding of significantly different values of *D* for the antiGFP-QD605 and nanoGFP-QD605 probes, we analyzed the mean square displacement as a function of time delay, computed from the antiGFP-QD605, nanoGFP-QD605, and nanoGFP-QD525 probe trajectories (see Materials and Methods). In Figure 2*G*, the population MSD curves are plotted for trajectories that were classified as synaptic (using the strictest Method 1). The short-time diffusion coefficient *D* adequately describes the motion during the first 1 s (Fig. 2*G*, inset), but at longer time scales there is a crossover to a slower diffusive mode, starting at MSD values <0.003 µm^2^ for all three probes. This indicates that the simple diffusion mode is limited to sub-100 nm distances, and that the *D* value cannot accurately predict how fast the probe traverses the whole synaptic area; rather, the inhomogeneities of the diffusion landscape limit the time to reach and cross the synaptic edge. We note, however, that the shortest residence times we observed with the nanoGFP-QD605 probe were several times lower than with the antiGFP-QD605 probe (Fig. 2*L*). This matches the expectation that for trajectories that are localized in the vicinity of the synaptic edge, the attempt rate for crossing the edge is controlled by the short-time diffusion coefficient *D*. Together, our data suggest that both QD605 probes have similar access to the synapse but that nanoGFP-QD605 probe is more mobile when compared with the antiGFP-QD605 probe and is thus most suitable for monitoring the surface mobility of different subtypes of NMDARs.

Last, we measured the *D* values for the extrasynaptic trajectories of nanoGFP-QD605-labeled YFP-GluN1-a subunits after blocking nonspecific protein-binding sites at the cell surface using either 1.25% casein or 1% BSA. We found no significant difference between the *D* values for extrasynaptic QD trajectories measured in the presence of casein and BSA [median *D* values: 0.16 µm^2^/s (casein); 0.24 µm^2^/s (BSA); Student's *t* test *t*_(1000)_ = 0.99, *p* = 0.34]. Given this observation, and given that casein had less of an effect on the hydrodynamic radius of both nanoGFP probes compared with BSA (Table 1), we used casein to block nonspecific protein-binding sites in our subsequent experiments.

### Surface localization and mobility of GFP-GluN2A, GFP-GluN2B, and GFP-GluN3A subunits in hippocampal neurons measured using the nanoGFP-QD605 probe

Next, we measured the surface localization and mobility of NMDARs in cultured hippocampal neurons expressing GFP-GluN2A, GFP-GluN2B, or GFP-GluN3A subunits. We chose these specific GFP-tagged GluN subunits for two reasons. First, these constructs have been used in many previous studies addressing both the trafficking and function of NMDARs (Pérez-Otaño et al., 2001; Luo et al., 2002; Kaniakova et al., 2012; Skrenkova et al., 2019). Second, our electrophysiological measurements in transfected HEK293 cells showed that the presence of the GFP tag does not affect the l-glutamate or glycine EC_50_ values measured for GluN1-4a/GluN2A and GluN1-4a/GluN2B receptors (Table 2); nor does it affect the EC_50_ values for glycine (Table 2) or the τ_des_ measured for GluN1-4a/GluN3A receptors (Table 3).

**Table 2.** Summary of EC_50_ measured for the indicated NMDAR subtypes

Receptor	Agonist	EC_50_ (µm)	*h*	*n*
GluN1/GFP-GluN2A	l-glutamate*	5.4 ± 1.1	1.2 ± 0.1	4
GluN1/GluN2A	l-glutamate*	5.3 ± 0.6	1.2 ± 0.1	8
GluN1/GFP-GluN2B	l-glutamate*	2.7 ± 0.4	1.3 ± 0.1	7
GluN1/GluN2B	l-glutamate*	1.9 ± 0.3	1.3 ± 0.1	9
GluN1/GFP-GluN2A	Glycine†	2.9 ± 0.4	1.6 ± 0.1	4
GluN1/GluN2A	Glycine†	2.1 ± 0.1	1.4 ± 0.0	4
GluN1/GFP-GluN2B	Glycine†	0.4 ± 0.1	0.9 ± 0.1	4
GluN1/GluN2B	Glycine†	0.4 ± 0.1	1.0 ± 0.1	5
GluN1/GFP-GluN3A	Glycine†	58.4 ± 14.8	1.0 ± 0.2	4
GluN1/GluN3A	Glycine†	41.6 ± 18.4	0.8 ± 0.2	6

Transfected HEK293 cells were recorded at a membrane potential of −60 mV using the whole-cell patch-clamp method, current responses were elicited by rapid application of ECS containing the following concentrations of l-glutamate and/or glycine [GluN1/GluN2A – EC_50_ for l-glutamate: glycine (100 μm), l-glutamate (0.3; 1; 3; 10; 30; 100; 300 μm); GluN1/GluN2B – EC_50_ for l-glutamate: glycine (100 μm), l-glutamate (0.3; 1; 3; 10; 30; 100; 300 μm); GluN1/GluN2A – EC_50_ for glycine: l-glutamate (300 μm), glycine (0.1; 0.3; 1; 3; 10; 30 μm); GluN1/GluN2B – EC_50_ for glycine: l-glutamate (300 μm), glycine (0.1; 0.3; 1; 3; 10; 30 μm); GluN1/GluN3A – EC_50_ for glycine: glycine (10; 30; 100; 300; 1000; 3000; 10,000 μm]. Electrophysiological data were fitted using Equation 3; EC_50_ values (in μm), Hill coefficients (*h*), and the numbers of cells analyzed (*n*) are shown; *p* > 0.05 for all EC_50_ values compared between untagged and GFP-tagged NMDARs subtypes (Student's *t* test). Data are the mean ± SEM.

*To measure the EC_50_ of l-glutamate, all solutions contained 100 μm glycine.

†To measure the EC_50_ of glycine, all solutions contained 300 μm l-glutamate.

**Table 3.** Summary of the time constant of desensitization in response to glycine measured for the indicated GluN1/GluN3A receptors

Receptor	Glycine (µm)	τ_des_ (ms)	*n*
GluN1/GFP-GluN3A	30	288.6 ± 15.7	5
GluN1/GFP-GluN3A	100	114.4 ± 5.6	5
GluN1/GFP-GluN3A	300	69.8 ± 3.4	5
GluN1/GFP-GluN3A	1000	51.1 ± 3.5	5
GluN1/GFP-GluN3A	3000	38.5 ± 4.8	5
GluN1/GFP-GluN3A	10 000	28.5 ± 2.5	5
GluN1/GluN3A	30	213.7 ± 23.6	6
GluN1/GluN3A	100	124.9 ± 12.0	6
GluN1/GluN3A	300	71.9 ± 11.1	6
GluN1/GluN3A	1000	37.1 ± 7.9	6
GluN1/GluN3A	3000	32.5 ± 4.9	6
GluN1/GluN3A	10 000	28.9 ± 2.7	6

Time constants of desensitization (τ_des_) calculated from the current responses of the indicated GluN1/GluN3A receptors expressed in HEK293 cells, elicited by the indicated glycine concentrations at a membrane potential of −60 mV. The obtained electrophysiological data were fitted using Equation 4; *p* > 0.05 when comparing untagged and GFP-tagged GluN1/GluN3A receptors at a given glycine concentration (Student's *t* test). Data are the mean ± SEM.

We used the nanoGFP-QD605 probe in the presence of casein to image the surface QD trajectories in neurons expressing GFP-GluN2A, GFP-GluN2B, or GFP-GluN3A subunits together with tdTomato-Homer1c (Fig. 3*A*). Our analysis of the tdTomato-Homer1c signal using Method 1 (Fig. 2*B*) showed that neurons expressing GFP-GluN2A and GFP-GluN2B subunits had similar synaptic regions ∼0.085 µm^2^ in area; in contrast, the synaptic region was significantly larger in neurons expressing GFP-GluN3A subunit (∼0.092 µm^2^; Fig. 3*B*). Next, we compared the distances between the QD trajectory points and the edge of the synaptic region. We found that the QD trajectories of both GFP-GluN2A-containing and GFP-GluN2B-containing NMDARs had similar distributions, with the main peak occurring near 0 nm (i.e., at the edge of the synaptic region). In contrast, the GFP-GluN3A-containing NMDARs had a clear rightward shift and a broader distribution, with a smaller peak occurring at ∼500 nm (Fig. 3*C*). Next, we calculated the localization density of QD trajectories; this approach normalizes the localization counts by the membrane area and provides a direct estimate of the surface density of QDs in the synaptic region. This analysis revealed that the GFP-GluN2A-containing and GFP-GluN2B-containing NMDARs had a distinct density peak located in the synaptic region at approximately −80 nm, whereas the GFP-GluN3A-containing NMDARs showed a rather flat distribution with no obvious peak (Fig. 3*D*). Most central localizations were detected at approximately −175 nm, and values of localization density in the synaptic region followed a subunit-dependent relationship (GFP-GluN2A > GFP-GluN2B > GFP-GluN3A). Next, we aimed to exclude the possibility that our findings regarding differences in localization density between GFP-GluN2-containing and GFP-GluN3A-containing NMDARs are because of the larger synaptic regions in neurons expressing the GFP-GluN3A subunit. Therefore, we compared the localization density between all QD trajectories of the GFP-GluN2A-containing NMDARs and QD trajectories of the GFP-GluN2A-containing NMDARs found around synaptic regions with an area ranging from 0.092 ± 0.041 µm^2^ (based on our data with the GFP-GluN3A subunit; Fig. 3*B*); this analysis revealed no obvious difference between the two conditions (Fig. 3*E*).

Our initial analysis of the *D* values—regardless of whether the QD trajectories were synaptic or extrasynaptic—showed a pronounced subunit dependence, with the following rank order: GFP-GluN2A < GFP-GluN2B < GFP-GluN3A (Fig. 3*F*). These *D* values acquired from all QD trajectories were derived from two different pools (i.e., synaptic and extrasynaptic); therefore, these two pools should be analyzed separately (Groc et al., 2006). Given the different ways in which synaptic regions are determined by various groups, in addition to the aforementioned method of thresholding the tdTomato-Homer1c signals (i.e., Method 1; Figs. 2*B*, 3*G*), we also included a 120-nm-wide annular region around the edge of the tdTomato-Homer1c signal (Method 2; Fig. 3*G*); this region is referred to in the literature as the perisynaptic region and preferentially contains GluN3A-containing NMDARs (Pérez-Otaño et al., 2006). In both of these methods, a QD trajectory was assigned to the synaptic pool if >50% of its QD points were present in the predefined synaptic region. Finally, as a third method we defined a given QD trajectory as synaptic if the average distance of its QD points was <550 nm from the center of the nearest synaptic region (Method 3; Fig. 3*G*).

We first calculated the percentage of synaptic QD trajectories for all three GFP-GluN-containing NMDARs using all three methods and observed a decrease in the percentage of synaptic QD trajectories using increasingly rigid definitions of synaptic regions—regardless of the GFP-GluN subunit—with the following rank order: Method 3 > Method 2 > Method 1 (Fig. 3*H*). In addition, we found that the percentage of synaptic QD trajectories was subunit dependent—regardless of the method used—(Fig. 3*H*). These findings showed a prominent subunit-dependent preference of the studied NMDARs (GFP-GluN2A > GFP-GluN2B > GFP-GluN3A) for synaptic versus extrasynaptic regions, both at the level of individual localizations (Fig. 3*D*) and of entire trajectories (Fig. 3*H*).

We next calculated the *D* values separately for the QD trajectories in synaptic and extrasynaptic regions as defined by each method. In the case of extrasynaptic QD trajectories, we observed similar *D* values for NMDARs containing the individual GFP-GluN subunits, independent of the method used to define the synaptic regions. Once again, we also observed a clear subunit dependence of the calculated *D* values, with the same rank order (GFP-GluN2A < GFP-GluN2B < GFP-GluN3A; Fig. 3*I–K*). In the case of synaptic QD trajectories, we measured higher *D* values for the GFP-GluN3A-containing NMDARs compared with both the GFP-GluN2A-containing and the GFP-GluN2B-containing NMDARs, independent of the method used to define the synaptic regions (Fig. 3*I–K*). Moreover, the calculated *D* values of the synaptic QD trajectories of the GFP-GluN3A-containing NMDARs decreased as the method used to select the synaptic regions increased in rigidity, with the following rank order: Method 1 < Method 2 < Method 3. Similarly, we found that the method used to define the synaptic region was important for calculating the *D* values of the synaptic QD trajectories of the GFP-GluN2A-containing and GFP-GluN2B-containing NMDARs, as we observed a significant difference when Method 3 was used (with GFP-GluN2A < GFP-GluN2B), but we found no difference using the more rigid definitions of synaptic regions in Method 1 and Method 2 (Fig. 3*I–K*). Together, these results indicate that the surface mobility of GFP-GluN3A-containing NMDARs is higher than the surface mobility of both GFP-GluN2A-containing NMDARs and GFP-containing, GluN2B-containing NMDARs; moreover, the method used to define the synaptic region plays an important role when calculating the *D* value for NMDARs containing GFP-GluN subunits labeled with the nanoGFP-QD605 probe.

**Figure 4.**
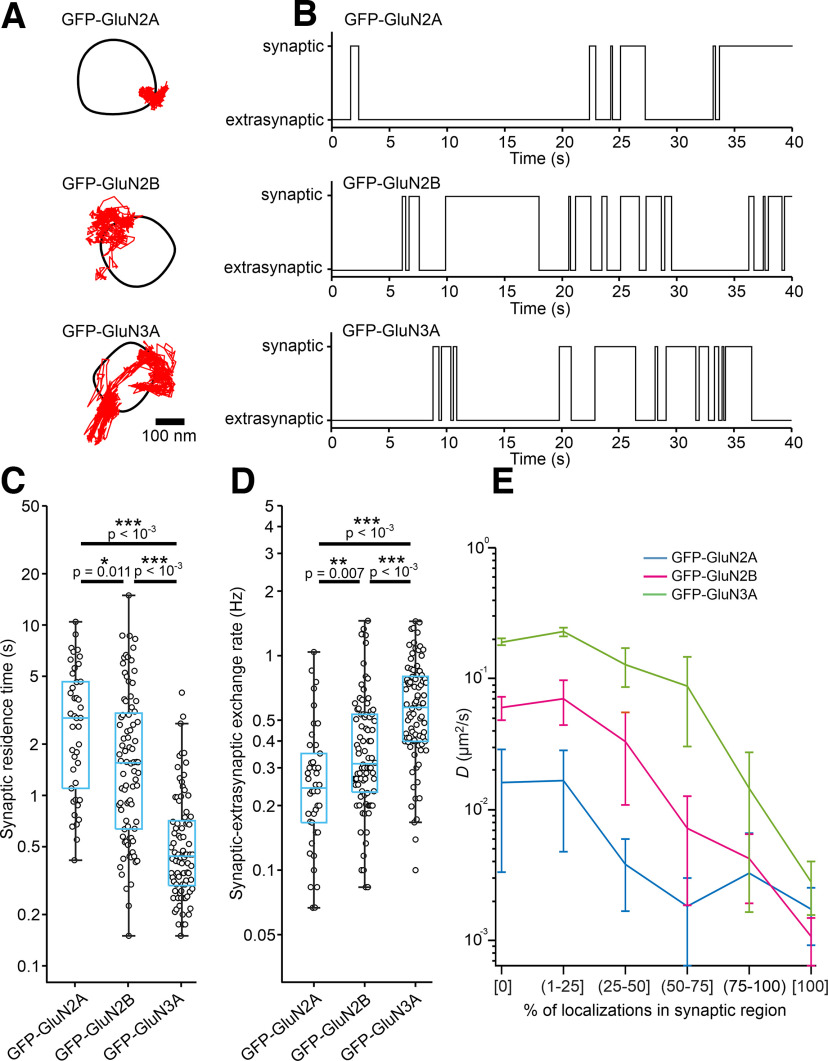
Detailed analysis of the QD trajectories measured in the synaptic and extrasynaptic regions in hippocampal neurons expressing GFP-GluN2A, GFP-GluN2B, or GFP-GluN3A subunits. ***A***, Representative surface QD trajectories (red) of NMDARs containing GFP-GluN2A, GFP-GluN2B, or GFP-GluN3A subunits showing the transitions between the synaptic and extrasynaptic regions (indicated by the black boundaries). ***B***, Corresponding filtered traces showing the indicated GFP-GluN subunits transitioning between the synaptic and extrasynaptic regions defined using Method 1. ***C***, Box plot summarizing the synaptic residence time calculated as the mean time that the QD-labeling indicated GFP-GluN subunits spent in the synaptic region; median values: 2.19 s (GFP-GluN2A); 1.35 s (GFP-GluN2B); and 0.46 s (GFP-GluN3A). ***D***, Box plot summarizing the synaptic–extrasynaptic exchange rate; note that only transitions >100 ms (i.e., ≥3 consecutive frames) were included in the analysis; median values: 0.25 Hz (GFP-GluN2A); 0.42 Hz (GFP-GluN2B); and 0.57 Hz (GFP-GluN3A). Note that ***C*** and ***D*** show log-transformed data with labels on the vertical axis kept as nontransformed reading times in seconds or frequencies in Hz; one-way ANOVA: *p* <10^−4^, *F*_(2,218)_ = 47.2 (for synaptic residency time); and *p* < 10^−4^, *F*_(2,218)_ = 22.75 (for exchange rate) followed by Bonferroni's multiple-comparisons test with *p*-values denoted in the plots. ***E***, Mean *D* values of the QD trajectories plotted against the percentage of time the trajectories spent inside the synaptic region; note that the *y*-axis is logarithmic and error bars represent the 95% confidence intervals.

Next, we examined whether synaptic residence time and/or the synaptic–extrasynaptic exchange rate is affected by the GluN subunit used. Therefore, we used Method 1 to determine whether the QDs were present in the synaptic or extrasynaptic region (Fig. 4*A*,*B*). We found that synaptic residence time was subunit dependent, with the following rank order: GFP-GluN2A > GFP-GluN2B > GFP-GluN3A (Fig. 4*C*); we also found that the synaptic–extrasynaptic exchange rate was subunit dependent—albeit in the reverse order as synaptic residence time—with the following rank order: GFP-GluN2A < GFP-GluN2B < GFP-GluN3A (Fig. 4*D*). These results are consistent with the observed differences in synaptic localization between GFP-GluN2A-containing, GFP-GluN2B-containing, and GFP-GluN3A-containing NMDARs (Fig. 3*D*).

Next, we sorted the QD trajectories into smaller pools based on the percentage of time (in 25% intervals) that they spent in the synaptic region (defined by Method 1) and calculated the mean *D* values. This approach revealed a trend common to all GFP-GluN subunits in which the higher the percentage of localization in the synaptic region, the smaller the *D* value (Fig. 4*E*). Furthermore, and consistent with our previous data, the *D* values were subunit dependent up to the −75% bin, with the following rank order: GFP-GluN3A > GFP-GluN2B > GFP-GluN2A (Fig. 4*E*); at >75%, we found no difference in *D* values among the three GFP-GluN subunits.

**Figure 5.**
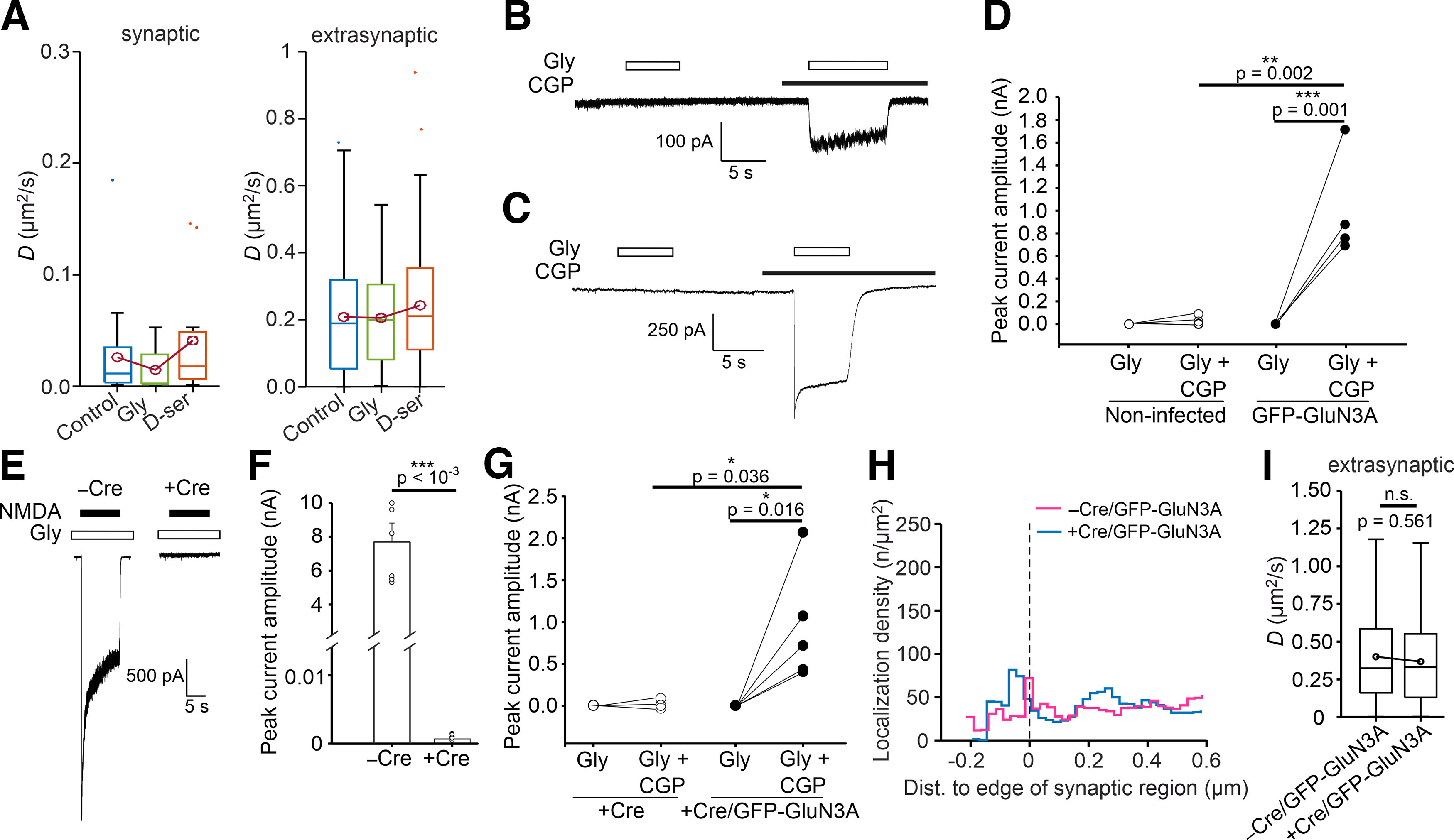
The presence of extracellular glycine, d-serine, or endogenous GluN2A and GluN2B subunits does not affect the synaptic or extrasynaptic QD trajectories of NMDARs containing GFP-GluN3A subunit. ***A***, Box plots summarizing the *D* values calculated from the QD trajectories in neurons expressing GFP-GluN3A subunit measured in the synaptic and extrasynaptic regions (defined using Method 1). Where indicated, glycine (Gly; 30 μm) or d-serine (D-ser; 30 μm) were added to the imaging solution; median *D* values [synaptic, extrasynaptic] (µm^2^/s): Ctrl [0.0011, 0.1889]; glycine [0.0179, 0.2112]; d-serine [0.0027, 0.1996]; K–W test: *p* = 0.283, *F*_(2,33)_ = 2.52 (for synaptic QD trajectories); *p* = 0.364, *F*_(2,237)_ = 2.023 (for extrasynaptic QD trajectories). ***B***, ***C***, Representative whole-cell voltage-clamp recordings of noninfected rat hippocampal neurons (***B***) and those infected with the GFP-GluN3A subunit (***C***); both were obtained at a membrane potential of −60 mV. Currents were elicited by application of 100 μm glycine (Gly; empty bar); where indicated, 0.5 μm CGP-78608 (CGP; black bar) was applied immediately before and during Gly application (Gly+CGP). ***D***, Summary of peak current amplitudes evoked by 100 μm glycine (Gly) or 100 μm glycine and 0.5 μm CGP-78608 (Gly+CGP) application in rat hippocampal neurons noninfected or infected with GFP-GluN3A subunit; Student's *t* test: *t*_(7)_ = 5.172 Gly versus Gly+CGP (GFP-GluN3A); *t*_(7)_ = 5.034 noninfected versus GFP-GluN3A (Gly+CGP), *p*-values are denoted in the figure (*n* ≥ 5 cells/group). ***E***, Representative whole-cell patch-clamp recordings of current responses evoked by rapid application of 1 mm NMDA (black bar), in the continuous presence of 20 μm glycine (Gly), in hippocampal neurons from cKO-GluN2A/GluN2B mice (DIV14), which were infected at DIV7 using lentiviruses encoding tdTomato-Homer1c (–Cre) or Cre-tdTomato-Homer1c (+Cre); measured at a membrane potential of −60 mV. ***F***, Summary of peak current amplitudes evoked by NMDA application in hippocampal neurons from cKO-GluN2A/GluN2B mice (–Cre or +Cre; shown in ***E***); Student's *t* test: *t*_(10)_ = 5.158; (*n* ≥ 5 cells/group). ***G***, Summary of peak current amplitudes evoked by 100 μm glycine (Gly) or 100 μm glycine and 0.5 μm CGP-78608 (Gly+CGP) application in hippocampal neurons from cKO-GluN2A/GluN2B mice infected with Cre-tdTomato-Homer1c (+Cre) or coinfected with Cre-tdTomato-Homer1c and GFP-GluN3A subunit (+Cre/GFP-GluN3A); measured at a membrane potential of −60 mV; Student's *t* test: *t*_(7)_ = 6.124 Gly versus Gly+CGP (+Cre/GFP-GluN3A); *t*_(7)_ = 2.595 +Cre versus +Cre/GFP-GluN3A (Gly+CGP), *p*-values are denoted in the figure (*n* ≥ 5 cells/group). ***H***, Histogram of the localization density of nanoGFP- QD605 localizations plotted against the distance to the edge of the synaptic region (indicated by the vertical dashed line at 0); *n* > 9813 localizations for the labeled GFP-GluN3A subunit per condition, measured in hippocampal neurons from cKO-GluN2A/GluN2B mice coinfected with GFP-GluN3A subunit and tdTomato-Homer1c (–Cre/GFP-GluN3A) or Cre-tdTomato-Homer1c (+Cre/GFP-GluN3A). ***I***, Box plots summarizing the *D* values calculated from the extrasynaptic QD trajectories in hippocampal neurons from cKO-GluN2A/GluN2B mice coinfected with GFP-GluN3A subunit and tdTomato-Homer1c (-Cre/GFP-GluN3A) or Cre-tdTomato-Homer1c (+Cre/GFP-GluN3A), measured in extrasynaptic regions [defined using Method 1; median *D* values (µm^2^/s): –Cre/GFP-GluN3A 0.331, +Cre/GFP-GluN3A 0.324; passed the D'Agostino-Pearson's normality test after log-transformation; Student's *t* test: *t*_(311)_ = 0.582, with *p*-values denoted in plots].

Ferreira et al. (2017) previously reported that the GluN1 subunit coagonists glycine and d-serine differentially regulate the surface mobility of both GluN2A-containing and GluN2B-containing NMDARs. We therefore examined whether either ligand affects the surface mobility of GluN3A-containing NMDARs. Similar to the protocol used by Ferreira et al. (2017), we added 30 μm glycine or d-serine to the imaging solution and recorded the movement of the nanoGFP-QD605 probe for 60 s in hippocampal neurons expressing both tdTomato-Homer1c and GFP-GluN3A subunits. Our analysis revealed no difference in *D* values between control conditions and the presence of either glycine or d-serine, regardless of whether we examined synaptic or extrasynaptic regions (Fig. 5*A*). A possible explanation of the previous experiment is that our infected neurons did not have functional GluN3A-containing NMDARs on their cell surface. Subsequent electrophysiological recordings showed that noninfected hippocampal neurons exhibited small but distinguishable current responses induced by 100 μm glycine in the presence of 0.5 μm CGP-78608 (which reduces GluN1-mediated desensitization and thus “unmasks” the GluN1/GluN3A receptors; Fig. 5*B*,*D*; Grand et al., 2018). Using rat hippocampal neurons infected with the GFP-GluN3A subunit, we observed increased amplitudes of current responses induced by 100 μm glycine in the presence of 0.5 μm CGP-78608 (Fig. 5*C*,*D*). In both noninfected and infected neurons, we observed no or negligible glycine-induced current responses in conditions without CGP-78608, likely because of strong desensitization of GluN3A-containing NMDARs (Fig. 5*B–D*). In summary, our electrophysiological experiments confirmed the presence of functional GFP-GluN3A-containing NMDARs on the surface of infected rat hippocampal neurons.

Although original studies suggested the presence of triheteromeric GluN1/GluN2/GluN3A receptors (Pérez-Otaño et al., 2001, 2016), recent data support the exclusive presence of diheteromeric GluN1/GluN3A receptors in mammalian neurons (Bossi et al., 2022). We next used hippocampal neurons from cKO-GluN2A/GluN2B mice to determine whether simultaneous KO of both GluN2A and GluN2B subunits alters the surface localization and mobility of the GFP-GluN3A subunit containing NMDARs. Our electrophysiological experiments showed that expression of Cre-tdTomato-Homer1c for 7 d completely eliminated NMDA-mediated current responses in DIV14-aged hippocampal neurons (Fig. 5*E*,*F*), confirming the complete KO of surface GluN1/GluN2 receptors. Further electrophysiological measurements from these hippocampal neurons coinfected with Cre-tdTomato-Homer1c and the GFP-GluN3A subunit revealed profound current responses induced by 100 μm glycine in the presence of 0.5 μm CGP-78608, but not by 100 μm glycine alone (Fig. 5*G*). Thus, our electrophysiological experiments showed that the infected GFP-GluN3A subunit is present on the neuronal surface even in the absence of GluN1/GluN2 receptors, likely in the form of functional diheteromeric GluN1/GluN3A receptors.

We next examined the surface mobility of GFP-GluN3A-containing NMDARs in hippocampal neurons from cKO-GluN2A/GluN2B mice coinfected with tdTomato-Homer1c or Cre-tdTomato-Homer1c using the nanoGFP-QD605 probe. This experiment showed that the localization density of the QD trajectories calculated by defining a concentric zone within and around the synaptic region with a thickness of 25 nm (Fig. 5*H*) as well as *D* values of the extrasynaptic QD trajectories (Fig. 5*I*) for the GFP-GluN3A-containing NMDARs did not differ between the neurons either expressing or lacking GluN2A and GluN2B subunits. These findings support the presence of only diheteromeric GluN1/GluN3A receptors in our cultured hippocampal neurons (see Discussion).

### Surface localization and mobility of GFP-GluN2A, GFP-GluN2B, and GFP-GluN3A subunit-containing NMDARs in hippocampal neurons measured using uPAINT with nanoGFP-ATTO647N probe

**Figure 6.**
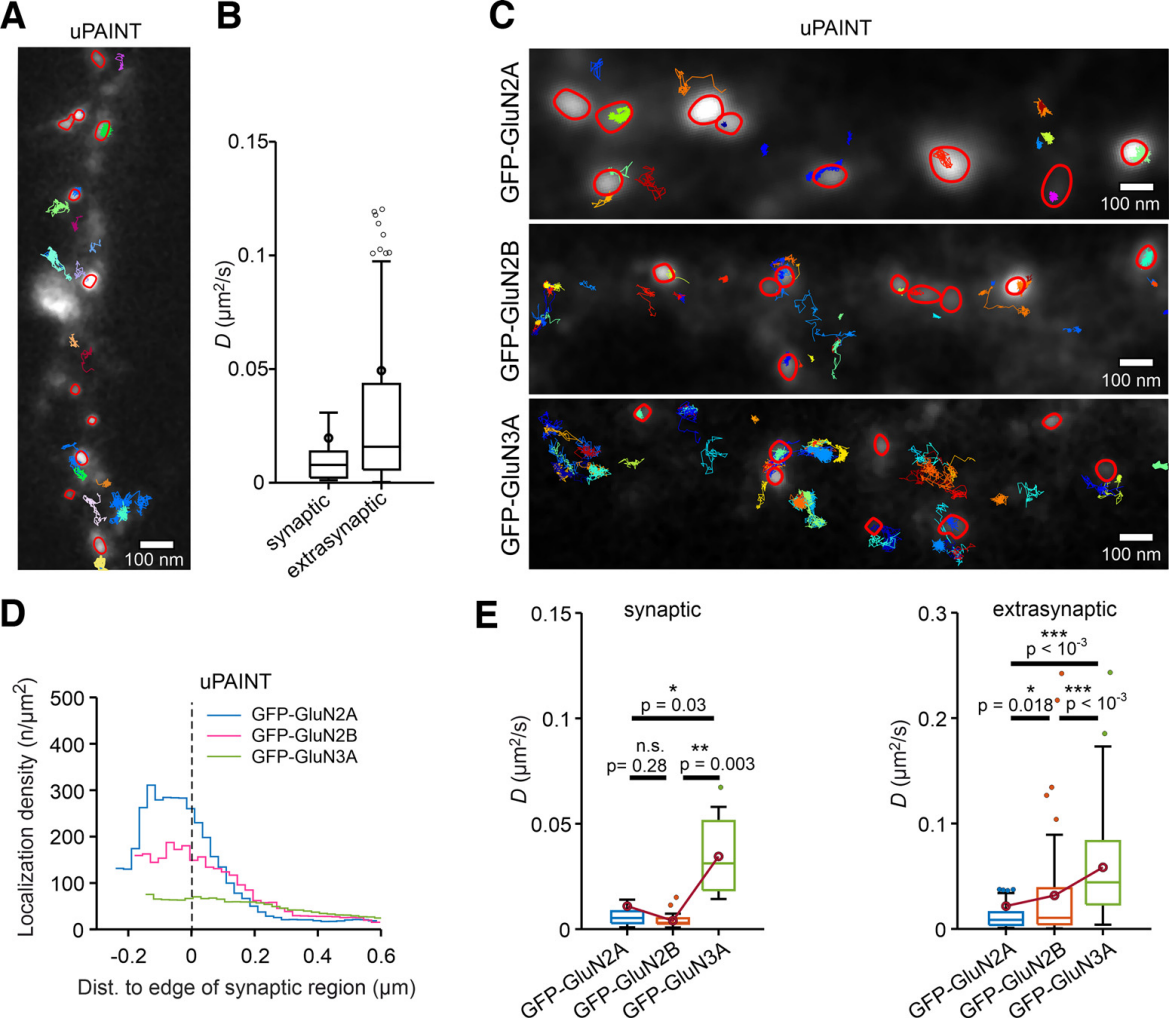
Surface mobility of YFP-/GFP-GluN subunit-containing NMDARs in hippocampal neurons measured by uPAINT using the nanoGFP-ATTO647N probe. ***A***, Examples of surface nanoGFP-ATTO647N trajectories (colored trajectories) measured in rat hippocampal neurons coexpressing YFP-GluN1-1a subunit and the synaptic marker tdTomato-Homer1c (gray) to define the synaptic regions (indicated by the red boundaries). The neurons were imaged for 5 × 60 s with a 50 ms interval between frames. ***B***, Box plots summarizing the *D* values derived by the linear fit of the MSD curves (see Materials and Methods); median *D* values [synaptic, extrasynaptic] (µm^2^/s): nanoGFP-ATTO647N [0.0079, 0.0159]. ***C***, Representative surface ATTO647N trajectories (colored trajectories) of GFP-GluN2A, GFP-GluN2B, or GFP-GluN3A-containing NMDARs in rat hippocampal neurons expressing tdTomato-Homer1c (gray). ***D***, Histogram of the localization density of ATTO647N localizations plotted against distance to the edge of the synaptic region (indicated by the vertical dashed line at 0); *n* > 34,000 ATTO647N localizations/group. Negative and positive distances indicate ATTO647N localizations inside and outside of the synaptic region, respectively. ***E***, Box plots summarizing the *D* values calculated from synaptic and extrasynaptic ATTO647N trajectories; median *D* values [synaptic, extrasynaptic] (µm^2^/s): GFP-GluN2A [0.0053, 0.0086]; GFP-GluN2B [0.0031, 0.0106]; GFP-GluN3A [0.0312, 0.0443]; K–W test: *p* = 0.004, *F*_(2,44)_ = 10.84 (for synaptic ATTO647N trajectories); *p* < 10^−3^, *F*_(2,337)_ = 126.7 (for extrasynaptic ATTO647N trajectories); followed by Bonferroni's multiple-comparisons test with *p*-values denoted in the figure.

Although commercially available QDs are commonly used to track the synaptic motility of both NMDARs and AMPARs (Groc and Choquet, 2020), their size, including the PEG linker, may restrict the receptor–nanobody–QD complex from fully entering into the synaptic cleft (Lee et al., 2017). Therefore, we next used the uPAINT method in combination with the nanoGFP-ATTO647N probe, first to examine the surface trajectories of YFP-GluN1-1a subunits in rat hippocampal neurons coexpressing tdTomato-Homer1c (Fig. 6*A*). This method allowed us to track the mobility of YFP-GluN1-1a subunits for an average of 7.2 ± 8.6 s (mean ± SD) and then to calculate *D* values, both in synaptic (defined by Method 1) and extrasynaptic regions (Fig. 6*B*). The obtained median *D* values were several-fold higher compared with those obtained with the nanoGFP-QD605 probe (Fig. 2*I*); the distribution of *D* values for the nanoGFP-QD605 probe was more asymmetric and skewed toward lower *D* values (compare box plots Figs. 2*I*, 6*B*). Note that the uPAINT analysis was based on considerably shorter trajectories, which may affect the direct comparison of *D* values between the nanoGFP-QD605 and nanoGFP-ATT647N probes (see Discussion).

Next, we compared the surface nanoGFP-ATTO647N trajectories of the NMDARs containing GFP-GluN2A, GFP-GluN2B, and GFP-GluN3A subunits in rat hippocampal neurons coinfected with Tomato-Homer1c (Fig. 6*C*). Our analysis of the localization densities calculated from the nanoGFP-ATTO647N trajectories showed that the NMDARs containing GFP-GluN2A and GFP-GluN2B subunits had a major density peak in the region of approximately −80 nm; in contrast, the GFP-GluN3A-containing NMDARs showed a broader distribution without a significant peak (Fig. 6*D*). Similar to the nanoGFP-QD605 probe, we observed subunit-dependent differences in the maximal values of the localization density within the synaptic region (GFP-GluN2A > GFP-GluN2B > GFP-GluN3A). Compared with the nanoGFP-QD605 probe, the nanoGFP-ATTO647N probe exhibited a lower localization density and a spatial profile with a slightly broader peak. The lower localization density reflects the methodological limitations of the uPAINT experiments; however, each bin in the peak region for GFP-GluN2A and GFP-GluN2B subunits (Fig. 6*D*, histogram) collects >1500 localizations, resulting in a well defined monophasic shape of the estimated density profile. The localization uncertainty of ∼16 nm of the nanoGFP-ATTO647N probe (compared with ∼5 nm for the nanoGFP-QD605 probe) could contribute to an apparent broadening of the localization density peak by up to ∼20 nm (see Discussion).

Next, we calculated the *D* values for NMDARs containing GFP-GluN subunits, both in synaptic (defined by Method 1) and extrasynaptic regions of rat hippocampal neurons. For extrasynaptic nanoGFP-ATTO647N trajectories, we observed a subunit-dependent relationship of *D* values (GFP-GluN2A < GFP-GluN2B < GFP-GluN3A; Fig. 6*E*). Furthermore, we observed that synaptic trajectories of the nanoGFP-ATTO647N probe showed higher *D* values for the GFP-GluN3A-containing NMDARs compared with both GFP-GluN2A-containing and GFP-GluN2B-containing NMDARs (Fig. 6*E*). These trends are consistent with our data obtained using the nanoGFP-QD605 probe, thus indicating that the size of the nanoGFP-QD605 probe is not a limiting factor in our studies with synaptic pools of GFP-tagged NMDARs (see Discussion).

### Surface localization of GFP-GluN2A, GFP-GluN2B, and GFP-GluN3A subunit-containing NMDARs in hippocampal neurons measured by dSTORM with the nanoGFP-AF647 probe

**Figure 7.**
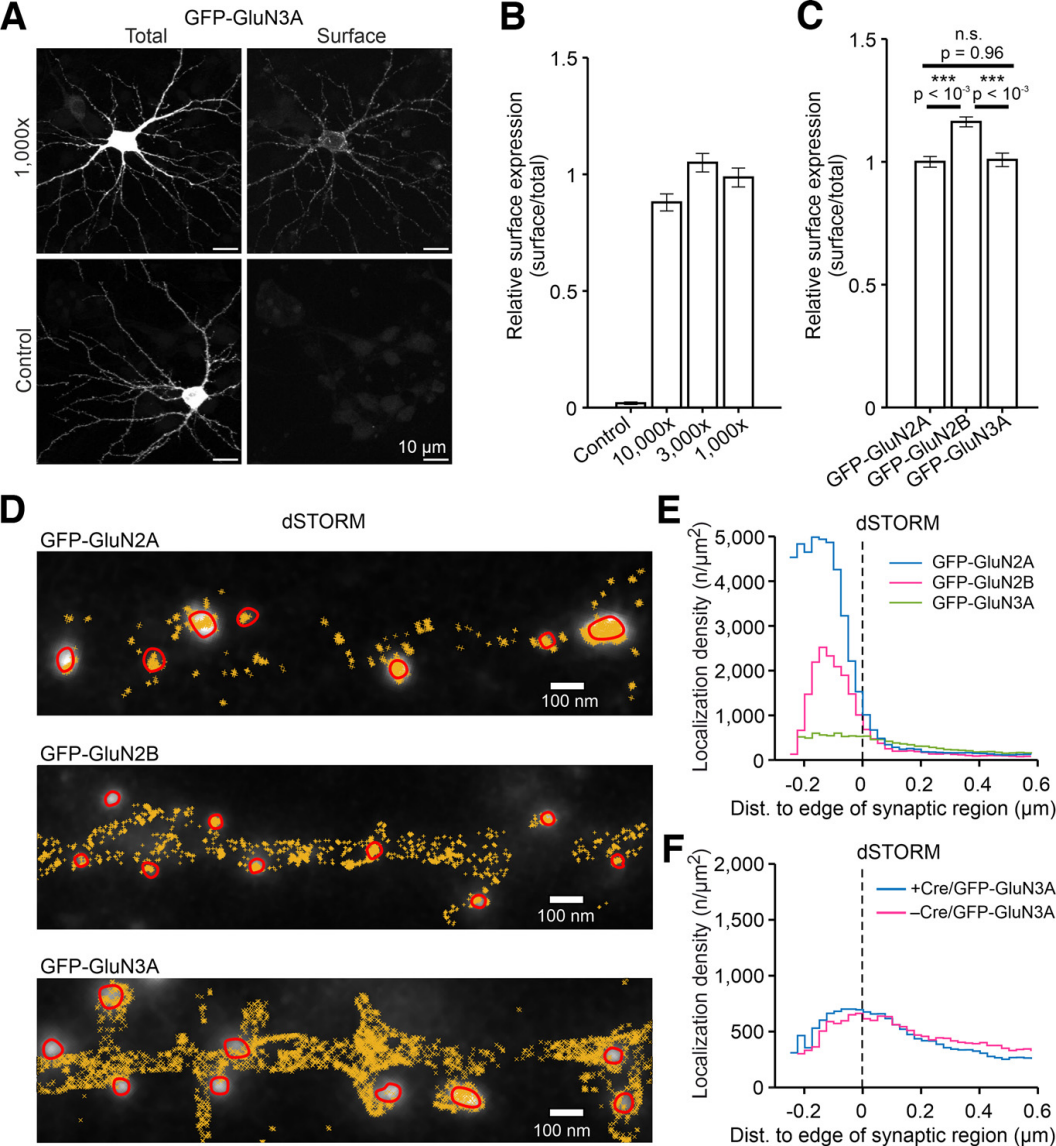
Surface localization of GFP-GluN-containing NMDARs in hippocampal neurons measured by dSTORM using the nanoGFP-AF647 probe. ***A***, Representative images of rat hippocampal neurons (DIV14) infected with GFP-GluN3A subunit; total (labeled using primary anti-GFP antibody and secondary anti-antibody conjugated with AF488) and surface (obtained using 1000-fold diluted nanoGFP-AF647 probe) signals are shown; negative control was labeled without addition of nanoGFP-AF647 probe. ***B***, Summary of relative surface expression signals obtained by labeling with differently diluted nanoGFP-AF647 probes (1,000×; 3,000×; 10,000×) as described in ***A***; measured in 10 μm segments of secondary or tertiary dendrites of rat hippocampal neurons infected with the GFP-GluN3A subunit (*n* ≥ 24 segments in ≥6 different cells/group). ***C***, Summary of relative surface expression of indicated GFP-GluN subunits measured in 10 μm segments of secondary or tertiary dendrites of infected rat hippocampal neurons, labeled as described in ***A*** (*n* ≥ 24 segments in ≥6 different cells/group); one-way ANOVA (*F*_(3,287)_ = 53.0405, *p* < 0.001 followed by Bonferroni's multiple-comparisons test with *p*-values denoted in the figure. ***D***, Selected dSTORM images of rat hippocampal neurons infected with GFP-GluN2A, GFP-GluN2B, or GFP-GluN3A subunits that were labeled with nanoGFP-AF647 probe. ***E***, Histogram of the localization density of NMDARs containing the GFP-GluN2A, GFP-GluN2B, and GFP-GluN3A subunits labeled with the nanoGFP-AF647 probe. The AF647 localizations are plotted against distance to the edge of the synaptic region, measured in rat hippocampal neurons coinfected with tdTomato-Homer1c (*n* > 161,793 nanoGFP-AF647 localizations/group). ***F***, Histogram of the localization density of AF647 localizations plotted against distance to the edge of the synaptic region, measured in hippocampal neurons from cKO-GluN2A/GluN2B mice coinfected with GFP-GluN3A subunit and tdTomato-Homer1c (–Cre/GFP-GluN3A) or Cre-tdTomato-Homer1c (+Cre/GFP-GluN3A; *n* > 12,998 nanoGFP-AF647 localizations/group).

Our microscopy experiments with the nanoGFP-QD605 and nanoGFP-ATTO647N probes showed a different distribution of the studied GFP-GluN subunits in synaptic regions; however, these data were obtained using sparsely and stochastically labeled NMDARs. Therefore, we further decided to use dSTORM in combination with a saturating concentration of the nanoGFP-AF647 probe on hippocampal neurons infected with GFP-GluN subunits. Using confocal microscopy, we first tested the optimal concentration of the nanoGFP-AF647 probe to achieve a fully saturating signal of surface NMDARs containing the GFP-GluN3A subunit in rat hippocampal neurons; total expression of the GFP-GluN3A subunit was examined using primary and secondary antibodies after fixation and permeabilization of neurons (Fig. 7*A*). These experiments showed that the 1000-fold-diluted nanoGFP-AF647 probe is suitable for further microscopy experiments because it labels the surface GFP-GluN3A subunit at a level similar to those with higher dilutions (i.e. 3000-fold and 10,000-fold; Fig. 7*B*). To examine the surface expression of individual GFP-GluN subunits in rat hippocampal neurons, we further labeled surface GFP-GluN2A, GFP-GluN2B, and GFP-GluN3A subunits using a saturating concentration of the nanoGFP-AF647 probe (diluted 1000-fold); total expression of the GFP-GluN subunits was studied using a combination of primary and secondary antibodies. Using confocal microscopy, we found that NMDARs containing GFP-GluN2A subunits were expressed on the surface of neurons at similar levels as NMDARs containing GFP-GluN3A subunits, whereas surface expression of NMDARs containing the GFP-GluN2B subunit was slightly increased (Fig. 7*C*).

Finally, we performed dSTORM imaging on rat hippocampal neurons coinfected with GFP-GluN2A, GFP-GluN2B, and GFP-GluN3A subunits (labeled with a 1000-fold-diluted nanoGFP-AF647 probe) and tdTomato-Homer1c (synaptic regions were defined by Method 1; Fig. 7*D*). We found that NMDARs containing GFP-GluN2A and GFP-GluN2B subunits had a major peak of their localization density in the region of approximately −100 nm, although the values of localization density of NMDARs containing GFP-GluN2A remained high up to approximately −200 nm. In contrast, NMDARs containing the GFP-GluN3A subunit showed a broader distribution with a less pronounced peak in the region of ∼0 nm (Fig. 7*E*).

Similar to our findings from QD tracking and uPAINT, with the dSTORM data, we observed prominent subunit-dependent differences in the peak values of localization density of nanoGFP-AF647 signals within synaptic regions of rat hippocampal neurons (GFP-GluN2A > GFP-GluN2B > GFP-GluN3A). We further compared the localization density of GFP-GluN3A subunit-containing NMDARs in hippocampal neurons from cKO-GluN2A/GluN2B mice coinfected with tdTomato-Homer1c or Cre-tdTomato-Homer1c. Our analysis showed a similar localization density of GFP-GluN3A subunit-containing NMDARs, independent of the presence of Cre recombinase (Fig. 7*F*). This indicates that the presence of endogenous GluN2A and GluN2B subunits does not alter the surface distribution of NMDARs containing the GFP-GluN3A subunit, which is consistent with our findings obtained using the nanoGFP-QD605 probe (Fig. 5*H*,*I*).

In summary, our experiments with all nanobody-based probes (1) demonstrate that our nanoGFP-QD605 probe can be used to study the surface localization and mobility of NMDARs, (2) reveal the importance of accurately defining the synaptic region to analyze the surface localization and mobility of NMDARs, and (3) are consistent with the observed subunit-dependent differences in the surface localization and mobility of NMDARs.

## Discussion

Our objective was to develop and characterize a probe suitable for studying the surface mobility of NMDARs. We chose to use nanoGFP-QD-based probes in neurons expressing YFP-GluN/GFP-GluN subunits for several reasons. First, the gene sequence for expressing the nanoGFP protein is publicly available, and the DNA expression vector can be obtained from Addgene. Second, nanoGFP has extremely high affinity for GFP (Kubala et al., 2010). Third, the size of nanoGFP is markedly smaller compared with conventional IgG antibodies (see Introduction). Fourth, the YFP-GluN/GFP-GluN constructs that we used are routinely used by the scientific community. Finally, both QD525 and QD605 are commercially available. Based on our experimental data, the published size of nanoGFP, and the predicted size of the used linker (∼1 nm), we estimate that the maximal sizes of our nanoGFP-QD525 and nanoGFP-QD605 probes are ∼21 and ∼25 nm, respectively.

Recent studies (Lee et al., 2017; Le et al., 2020) evaluated the synaptic accessibility for AMPARs labeled with quantum dots of distinct sizes as well as with fluorescent dyes, and found that the fraction of labeled receptors localized in the synaptic cleft region dropped strongly for the largest probes. An analogous comparison of probes of different sizes has not been previously reported for NMDARs. Our analysis of QD trajectories measured in neurons expressing the YFP-GluN1-1a subunit did not show major differences in synaptic cleft accessibility when using the nanoGFP-QD-based probes compared with the antiGFP-QD605 probe. However, with the nanoGFP-QD-based probes we observed significantly higher *D* values for both the synaptic and extrasynaptic pools, as well as a steeper increase of *D* as a function of increasing distance from the synaptic region edge. Moreover, the *D* values we obtained with the nanoGFP-QD-based probes were closer to the *D* values obtained for the YFP-GluN1-1a subunit labeled using the nanoGFP-ATTO647N probe, in which the QD was replaced by a smaller fluorescent dye. Given the higher localization accuracy of the nanoGFP-QD605 probe compared with the nanoGFP-QD525 probe, we used the nanoGFP-QD605 probe for our detailed analysis of the surface mobility of NMDARs containing GFP-GluN subunits.

Our results regarding the *D* values obtained for the extrasynaptic QD trajectories of NMDARs containing GFP-GluN2 subunits (with GFP-GluN2A < GFP-GluN2B) are consistent with a previous study using classic IgG antibodies, which showed that extrasynaptic GluN2A-containing NMDARs had a lower *D* value compared with GluN2B-containing NMDARs (Groc et al., 2006). We further focused on establishing the ideal parameters for analyzing the QD trajectories of three GFP-tagged GluN subunits using a precise definition of synaptic and extrasynaptic regions based on the tdTomato-Homer1c signal. First, we found that using Method 3 resulted in a clear difference in *D* values between GFP-GluN2A-containing and GFP-GluN2B-containing NMDARs in both the synaptic and extrasynaptic pools; in contrast, we found no difference in *D* values between synaptic GFP-GluN2A-containing and GFP-GluN2B-containing NMDARs when we defined the synaptic regions using either Method 1 or Method 2. Thus, accurately defining the synaptic region is essential for properly analyzing the surface QD trajectories of NMDARs containing GFP-GluN subunits. Second, our analysis revealed clear subunit-dependent differences in synaptic residence time and the synaptic–extrasynaptic exchange rate, consistent with previous findings (Groc et al., 2006). Third, we found that the *D* values differed between the GFP-GluN2A-containing and GFP-GluN2B-containing NMDARs only when we included QD trajectories that were present in the synaptic region <75% of the time.

Our detailed analysis of the QD trajectories of GFP-GluN3A-containing NMDARs revealed that these receptors have (1) the lowest synaptic residence time and (2) the highest synaptic–extrasynaptic exchange rate among all three GFP-GluN-containing NMDARs. This is consistent with previous studies showing the preferential localization of GluN3A-containing NMDARs in both perisynaptic and extrasynaptic regions (Pérez-Otaño et al., 2006). This finding may have physiologically relevant implications. For example, the high surface mobility of GluN3A-containing NMDARs may allow for a rapid change in the number of synaptic and/or perisynaptic NMDARs during the critical period of synapse maturation (Rakic et al., 1986; Holtmaat et al., 2005; Kehoe et al., 2014; Crawley et al., 2022). Moreover, we observed that the higher surface mobility of GFP-GluN3A-containing NMDARs and their relatively low preference for synaptic localization do not depend on the presence of endogenous GluN2A and GluN2B subunits. Thus, our data are consistent with the conclusion that only diheteromeric GluN1/GluN3A receptors, but not triheteromeric GluN1/GluN2/GluN3A receptors, are expressed in mammalian neurons (Bossi et al., 2022).

We cannot exclude the possibility that the presence of the ∼27 kDa extracellular GFP tag affected the surface mobility of GFP-GluN2-containing NMDARs in the synaptic region; however, this is unlikely given that we found that the GFP-GluN3A-containing NMDARs had higher *D* values in both the extrasynaptic and synaptic regions, regardless of the method used to define the synaptic region. When we focused on the synaptic regions, we found that the calculated tdTomato-Homer1c signal area (on the order of 0.8–0.9 µm^2^) was consistent with previous studies (Dani et al., 2010; Tao-Cheng et al., 2014), although they were a slightly (∼8%) larger in neurons expressing the GFP-GluN3A subunit compared with neurons expressing either GFP-GluN2A or GFP-GluN2B subunits. We cannot rule out the possibility that our findings were influenced by changes in synaptic region and/or shapes of dendritic spine caused by overexpression of GFP-GluN subunits, as previously reported for the GluN3A subunit (Roberts et al., 2009; Kehoe et al., 2014). Therefore, future studies dealing with the surface mobility of NMDARs should also aim to incorporate structural information about the shape of dendritic spines during the imaging of live neurons.

A limitation of this study is that we defined the synaptic regions using 2D imaging; however, when analyzing a sufficient number of synapses and assuming that the orientation of the synapses is random, this approach should be valid and has been used previously (Groc et al., 2006; Ferreira et al., 2017). Similarly, trajectory tracking and localization were performed in 2D. We partially overcame this limitation by tracking secondary and tertiary dendrites with smaller diameters and selecting regions for subsequent analysis where QDs were in focus. Because of the tilted orientation and curvature of the membrane, the diffusion coefficients calculated from the projected trajectories underestimate the true value of *D*. The analysis of Renner et al. (2011) quantified this effect for tubular membranes, based on a dimensionless parameter that depends on *D*, the acquisition time, and the cylinder diameter. Evaluating this parameter for our datasets (Renner et al., 2011, their Fig. 1E) implies that the true *D* values were on average underestimated by 25% for the slower moving receptors and by ∼40% for the fastest receptors. This does not invalidate our conclusions, and in fact indicates that the distinctions we found among trajectory groups would be even more pronounced if expressed in terms of the true *D* values.

Using nanoGFP-QD605 and nanoGFP-ATTO647N probes, we observed a steeper increase in localization density toward the synapse for the NMDARs containing the GFP-GluN2A subunit compared with the GFP-GluN2B subunit; this subtype-dependent difference was even more apparent with the use of the nanoGFP-AF647 probe in combination with dSTORM. For the GFP-GluN2A-containing NMDARs, we observed a visible decrease in localization density in the central region of the synaptic region using nanoGFP-QD605 and nanoGFP-ATTO647N probes but not using the nanoGFP-AF647 probe. On the other hand, compared with GFP-GluN2A-containing NMDARs, GFP-GluN2B-containing NMDARs showed a reduced local density in the synaptic region using all three nanoGFP probes. This suggests that both nanoGFP-QD605 and nanoGFP-ATTO647N probes have limitations in accessing the center of the synaptic cleft. Since the nanoGFP-AF647 probe has a similar size to the nanoGFP-ATTO647N probe, it is unlikely that the size of the nanoGFP-ATTO647N probe was a limiting factor in accessing the center of the synaptic cleft.

**Figure 8.**
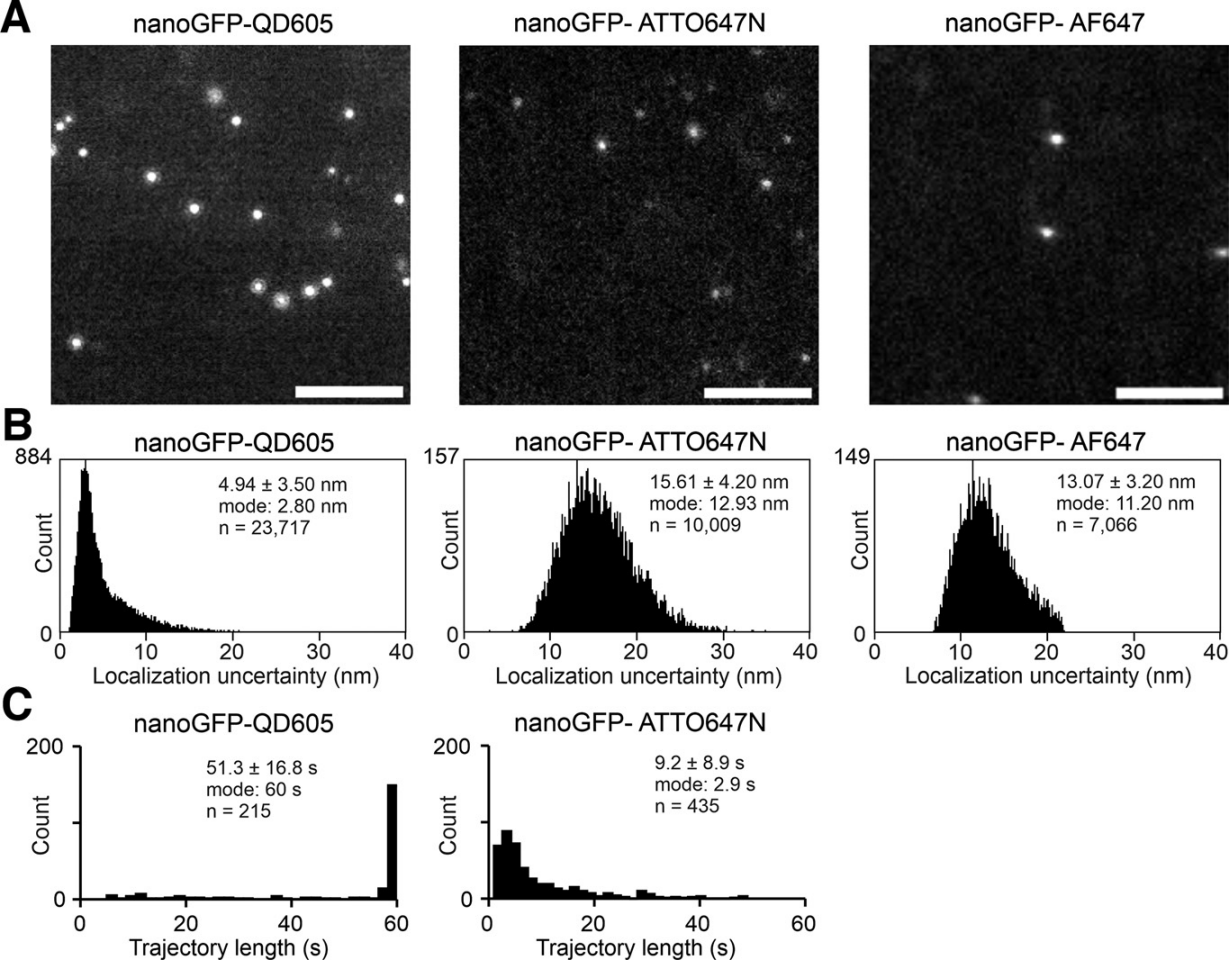
Comparison of nanoGFP-QD605, nanoGFP-ATTO647N, or nanoGFP-AF647 probes in respect of localization accuracy and/or effective lifetime for surface tracking of NMDARs. ***A***, Representative single frames of NMDARs containing GFP-GluN2A subunit stained by nanoGFP-QD605 or nanoGFP-ATTO647N probes for surface mobility/uPAINT and by nanoGFP-AF647 probe for dSTORM imaging. Scale bar, 6 µm. ***B***, Histogram of localization uncertainty from corresponding images of ***A***; localizations were detected in the representative area (20 µm^2^) with ImageJ plugin ThunderSTORM from multiple consecutive frames to reach *n* > 5000 localizations for each condition; probes were detected with the same detection parameters (B-spline wavelet filter: Order 3, scale 2.0; approximate localization, local maximum; threshold, 2 × background; connectivity, 8 neighborhoods; subpixel localization, PSF-integrated Gaussian; fitting radius, 3 px; fitting method, weighted least squares; initial σ, 1.6 px; multiemitter fitting, false); insets represent the average uncertainty (mean ± SD), the most often occurring uncertainty (mode), and the number of localizations (*n*); note that acquisitions of all three probes were performed with different microscopes. ***C***, Lengths of synaptic trajectories (defined by Method 1) recorded for GFP-GluN2A-containing NMDARs were used to compare the effective lifetime of nanoGFP-QD605 or nanoGFP-ATTO647N probes for surface tracking; histograms show the lengths of trajectories in seconds (s) recorded with indicated nanoGFP-QD605 or nanoGFP-ATTO647N probes; insets represent the average duration (mean ± SD), the most often occurring length (mode), and the number of trajectories (*n*).

The analysis of the level of uncertainty using the images of rat hippocampal neurons infected with the GFP-GluN2A subunit showed that the nanoGFP-QD605 probe had excellent localization accuracy (∼6 nm) and a virtually unlimited lifetime (Fig. 8*A*,*B*). The nanoGFP-ATTO647N probe, although smaller compared with the nanoGFP-QD605 probe, had reduced localization accuracy (∼16 nm; Fig. 8*A*,*B*) and limited lifetime (<10 s; Fig. 8*C*). Interestingly, the nanoGFP-AF647 probe, which also benefits from a smaller size, achieved in combination with dSTORM a similar localization accuracy (∼13 nm) as uPAINT (Fig. 8*A*,*B*). In summary, we conclude that both nanoGFP-QD605 and nanoGFP-ATTO647N probes are suitable for studying the surface mobility of NMDARs, but their methodological limitations should be considered. The experimental strategy described here can be developed further to study the effects of endogenous NMDAR ligands, pharmacological modulators of NMDARs such as the Food and Drug Administration-approved open-channel blockers ketamine and memantine (Song et al., 2018; Zhang et al., 2021), and pathogenic mutations in GluN subunits (e.g., the *ClinVar* database; https://www.ncbi.nlm.nih.gov/clinvar/), thus providing a deeper understanding of the mechanisms that regulate the surface mobility of specific NMDAR subtypes.
